# Land use change and carbon emissions of a transformation to timber cities

**DOI:** 10.1038/s41467-022-32244-w

**Published:** 2022-08-30

**Authors:** Abhijeet Mishra, Florian Humpenöder, Galina Churkina, Christopher P. O. Reyer, Felicitas Beier, Benjamin Leon Bodirsky, Hans Joachim Schellnhuber, Hermann Lotze-Campen, Alexander Popp

**Affiliations:** 1 Potsdam Institute for Climate Impact Research (PIK), Member of Leibniz Association, P.O.Box 60 12 03, 14412 Potsdam, Germany; 2 Humboldt University of Berlin, Department of Agricultural Economics, Unter den Linden 6, 10099 Berlin, Germany; 3 World Vegetable Center, P.O. Box 42, Shanhua, Tainan, 74199 Taiwan

**Keywords:** Climate-change mitigation, Forestry

## Abstract

Using engineered wood for construction has been discussed for climate change mitigation. It remains unclear where and in which way the additional demand for wooden construction material shall be fulfilled. Here we assess the global and regional impacts of increased demand for engineered wood on land use and associated CO_2_ emissions until 2100 using an open-source land system model. We show that if 90% of the new urban population would be housed in newly built urban mid-rise buildings with wooden constructions, 106 Gt of additional CO_2_ could be saved by 2100. Forest plantations would need to expand by up to 149 Mha by 2100 and harvests from unprotected natural forests would increase. Our results indicate that expansion of timber plantations for wooden buildings is possible without major repercussions on agricultural production. Strong governance and careful planning are required to ensure a sustainable transition to timber cities even if frontier forests and biodiversity hotspots are protected.

## Abstract (web summary)

Wood used in construction stores carbon and reduces the emissions from steel and cement production. Transformation to timber cities while protecting forest and biodiversity is possible without significant increase in competition for land.

## Introduction

In 2020, more than half of the global population lived in cities^1^. According to the Shared Socioeconomic Pathway 2 (SSP2) scenario, the global share of the population living in urban areas could rise to 80% by 2100 (92% in SSP1 scenario and 58% in SSP3 scenario)^1^. By the middle of this century, the newly built infrastructure (including new urban housing) may exceed the infrastructure being built since the beginning of industrialization^2^. Conventional buildings today are mostly built using steel and cement. Production of traditional building materials causes substantial anthropogenic CO_2_ emissions (e.g., due to carbonate calcination, electricity use, and fuel consumption from cement and steel production).

In 2020, raw material production for conventional buildings caused roughly 10% of the global greenhouse gas (GHG) emissions^3^, stemming from cement (1.48 Gt CO_2_e ^3^) as well as iron and steel (3.55 Gt CO_2_e ^3^) production. By 2100, about 30% of the CO_2_ emissions from concrete and mortar are expected to be again reabsorbed from the atmosphere through carbonation of hydrated cement products (concrete and mortar)^4,5^. Nevertheless, cement and steel production would still stay a net carbon emission source^6^. Continuous use of conventional building materials for future infrastructure development could claim 35–60% of the remaining carbon budget associated with limiting the global temperature increase to below 2 ^°^C^7^.

New and improved housing will be needed to accommodate the influx of new inhabitants into cities. Using engineered wood for constructing buildings can help to avoid emissions associated with conventional building materials. Wood is a renewable resource that usually carries the lowest carbon footprint of any comparable, first-time use, and building material^8^. Moreover, the carbon stored in wood, which was absorbed from atmospheric CO_2_ via photosynthesis, is partly preserved when the wood is used as a building material, making it a long-term carbon sink.

Wood is produced by harvesting forest plantations or natural forests (primary and secondary forests). In 2020, the plantation area was 132 Mha (i.e., 8% of global cropland area (1595 Mha) and only 4% of global natural forest area (3629 Mha)), but it likely contributed more than 33% to global industrial roundwood production^9^. In the mid to long term, highly productive plantations could therefore increase wood production while alleviating harvest pressure from natural forests under strict biodiversity and land protection regimes^10^. Wood needed for future timber constructions can come from increasing forest harvest from managed forest plantations and natural forests, redirecting existing wood uses, or establishing new forest plantations (which can be harvested at maturity in the future but provide wood from intermediate thinning until then)^6,9,11^.

Increasing forest harvest levels have negative impacts on biodiversity^12–14^. Currently, harvested wood is already used for other purposes and not necessarily available nor of adequate quality to be used as engineered wood. Furthermore, establishing new plantations has both land-use implications^11^ (in terms of competition for land) and negative biodiversity impacts^15^ when natural ecosystems are replaced. Therefore, the question of where to source the wood for the construction of timber cities is crucial.

Our study is the first to analyze the impacts of a large-scale transition to timber cities on land use, land-use change emissions, and long-term carbon storage in harvested wood products (HWP). A recent study^6^ quantified the building sector side of avoided carbon emissions when using timber as construction material. While that study highlights the mitigation potential of using engineered wood as construction material, it assumes that the increased demand for construction-grade engineered wood can potentially be supplied from the world’s forests based on historical trends and published projections of future biomass availability. Yet, especially the literature on the latter is scarce—relying solely on a rather old assessment of plantation change^16^ and a single-model global study using a Land-Surface Model with forest management. This is associated with considerable uncertainties since one generic forest harvesting scheme is applied globally^17^ and to a large extent ignores the effects of increasing timber demand on land-use dynamics. These assumptions about future biomass availability do not account for (1) the wood removal capacity from the world’s forests under land protection policies in a single integrated land-use modeling framework, (2) competition for limited land resources, (3) potential emissions due to forest harvesting and land-use change, and, (4) forest regrowth along with only partial accounting of carbon stored in harvested wood products.

To address these knowledge gaps, our central research questions are: where and in which way could an additional high demand for wooden construction material be produced, and what are the consequences in terms of direct and indirect CO_2_ emissions. This directly relates to the question of how competition for limited land resources evolves when timber production increases, and whether the production of additional timber (as engineered wood) for building purposes is within the forest use constraints posed by ambitious nature conservation and land protection targets. We also analyze the net climate benefit of timber-based buildings in comparison to traditional ways of constructing future buildings.

In this study, we calculate demand for engineered wood^18^ for the construction of wooden buildings based on the amount of new population influx into cities after 2020. We analyze four scenarios based on a recent study^6^—(1) “business as usual” (BAU) where no wooden buildings will be constructed for new urban dwellers, (2) “10% timber” (10 pc) where 10% of new urban population will live in wooden buildings, (3) “50% timber” (50 pc) where 50% of new urban population will live in wooden buildings; and (4) “90% timber” (90 pc) where 90% of new urban population will live in wooden buildings.

Here, we use a global multi-regional open-source land-system model MAgPIE^19,20^ (see “Methods”) to assess the future land-use and GHG consequences of using engineered wood predominantly as a building material. The model captures competition for land between agriculture and forestry and accounts for land-related GHG emissions. As such, it allows us to compare long-term carbon storage in HWP with emissions from the production of raw materials needed for constructing conventional buildings in the future. The future engineered wood demand scenarios for the period 2020–2100 are based on the trajectories of urban population growth according to SSP scenarios, namely SSP1 characterizing a world progressing towards sustainability, with the rapid development of low-income countries, SSP2 in which social, economic, and technological trends largely develop similar to historical patterns and a strongly fragmented world characterized by a high level of poverty in SSP3^1^. Future engineered wood demand is added as an additional constraint in the model, next to the demand for food, feed, bioenergy, and roundwood, which are based on SSP-specific population and income trajectories.

## Results

### Sourcing wood

**Fig. 1:**
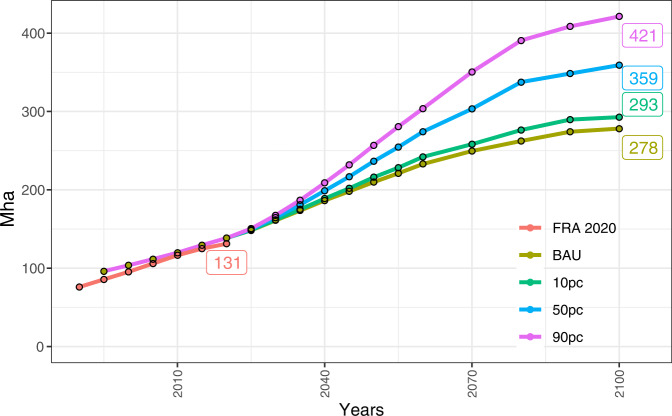
Evolution of the global forest plantation area between 1995 and 2100 in an SSP2 world. Business-as-usual (BAU) scenario is compared to, the 10%, 50%, and 90% (10 pc, 50 pc, 90 pc) engineered wood demand scenarios where 10%, 50%, or 90% of the new urban population will live in wooden buildings, respectively. Scenario projections are corroborated until 2020 with estimates from the Forest Resources Assessment (FRA) report 2020^21^. The numbers in the box are forest plantation areas (Mha) in 2100 for MAgPIE simulations and in 2020 for the reported value by the FRA.

For 2020, MAgPIE projects a global forest plantation area of 137 Mha, which compares well with data from FAOSTAT^21^. By the end of this century, the global plantation area is projected at 276 Mha in the BAU scenario, 291 Mha in the 10 pc scenario, 360 Mha in the 50 pc scenario, and 425 Mha in the 90 pc scenario, as shown in Fig. 1. Our future projections highlight that forest plantation areas would need to expand by more than 100% in 2100 compared to 2020 in the BAU scenario even without additional construction wood demand. To meet a considerable portion of engineered wood demand in the future, forest plantation areas would need to expand by more than 200% in the 90 pc scenario in 2100 compared to 2020. Roundwood (industrial roundwood and wood fuel) demand is the same across all scenarios. Therefore, the additional plantation area in the engineered wood demand scenarios is purely influenced by the higher demand for engineered wood. Compared to the historically available data^21^, the modeled value for forest plantations needed by 2100 for the high-end engineered wood demand scenario (90 pc) is on the higher end of observed trends. In the past decade, ca. 2 Mha of forest plantations were added annually. Modeled forest plantations of 425 Mha for the 90 pc scenario by 2100 result in an average rate of 3.6 Mha of newly established plantations annually which is on the higher end of past trends. These results should be interpreted within the context of assumptions made regarding the forest-land dynamics in our model (exclusion of pests, diseases, and forest fires, see “Methods”).

### Land-use change

**Fig. 2:**
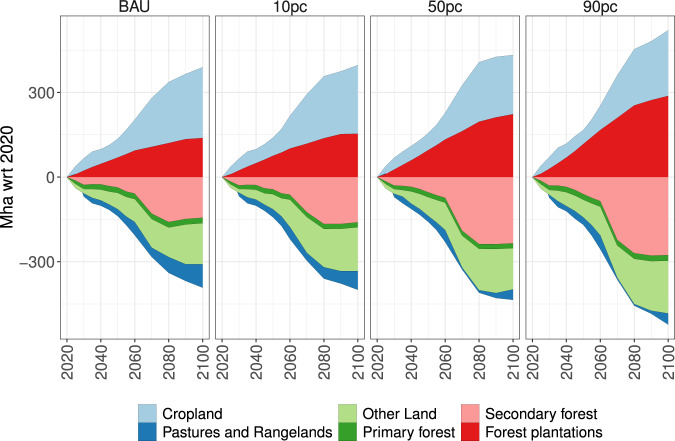
Comparitive changes in global land use between 2020 and 2100 (with respect to 2020). Changes in global land use are shown for cropland, pastures and rangelands, other land, primary forest, secondary forest, and forest plantations. Positive numbers represent an expansion of the land area for the indicated land-use type compared to 2020, and negative numbers represent a reduction of land area for the indicated land-use type compared to 2020. As the geographical land area is constant, the sum of positive changes (expansion) and negative changes (reduction) is always zero i.e., an expansion of a particular land-use type always comes at the cost of a reduction of another land-use type.

The land-use changes resulting from our three additional engineered wood demand scenarios (10 pc, 50 pc, and 90 pc) in terms of global dynamics for cropland, pasture, forest plantations, natural forests (primary forest and secondary forest) and non-forest natural vegetation (other land) throughout the 21st century are shown in Fig. 2 and Supplementary Fig. 6. In all scenarios, cropland and forest plantations expand at the cost of unprotected natural forests and “other land” (a land classification in MAgPIE which includes non-forest natural vegetation). In line with the high demand for engineered wood in regions with high (urban) population growth (e.g., Sub-Saharan Africa, see Supplementary Figs. 1,  2,  3, and Supplementary Fig. 6), these regions experience stronger land-use change compared to regions with low engineered wood demand (e.g., Europe, see Supplementary Figs. 1,  2,  3, and  6).

We estimate that a doubling of land-use intensification, driven by investments in yield-increasing technological change, between 2020 and 2100 is needed across all scenarios to increase the productivity of agricultural land, as plantation forestry and agriculture compete simultaneously for limited land resources (Supplementary Fig. 8). Investments in yield-increasing technological change (measured as Tau factor in MAgPIE^11,22^) are similar across the scenarios, not only on an aggregated global scale but also on a regional scale (Supplementary Fig. 8). Even though competition for land is not evident on the global or regional level, such competition could still exist on a finer spatial scale (Supplementary Fig. 13). The need for yield-increasing investments due to competition for land could also result in changes in agricultural land-use patterns due to geographical specialization in agricultural production^11^ (Supplementary Fig. 13).

With cropland and forest plantations expanding into natural forests, non-forest natural vegetation, and pasture land, mainly the tropics lose vast areas of unprotected primary and secondary forests (Supplementary Fig. 6). Cropland expansion is not hugely influenced by higher engineered wood demand, as the area expanded in the baseline scenario (BAU) is not drastically different from the higher engineered wood demand scenarios (Fig. 2 and Supplementary Fig. 8). Moreover, the agricultural commodity price index derived from MAgPIE is similar across all scenarios (Supplementary Fig. 7), which further indicates that wood can be produced for timber cities without drastic changes in agricultural prices.

Even though additional engineered wood demand for construction purposes can be met by utilizing forest plantations, this would result in a lot of new forest plantations being established on existing unprotected natural forests (Supplementary Figs. 4, 5) and non-forest natural vegetation. Natural forests and non-forest natural vegetation can, in principle, be converted to agricultural land or forest plantations, as long as the land protection and biodiversity constraints in MAgPIE are not violated. This encroachment in natural forests is feasible in MAgPIE but in reality, might entail losses in biodiversity and soil carbon.

### Cumulative land-use change emissions

**Fig. 3:**
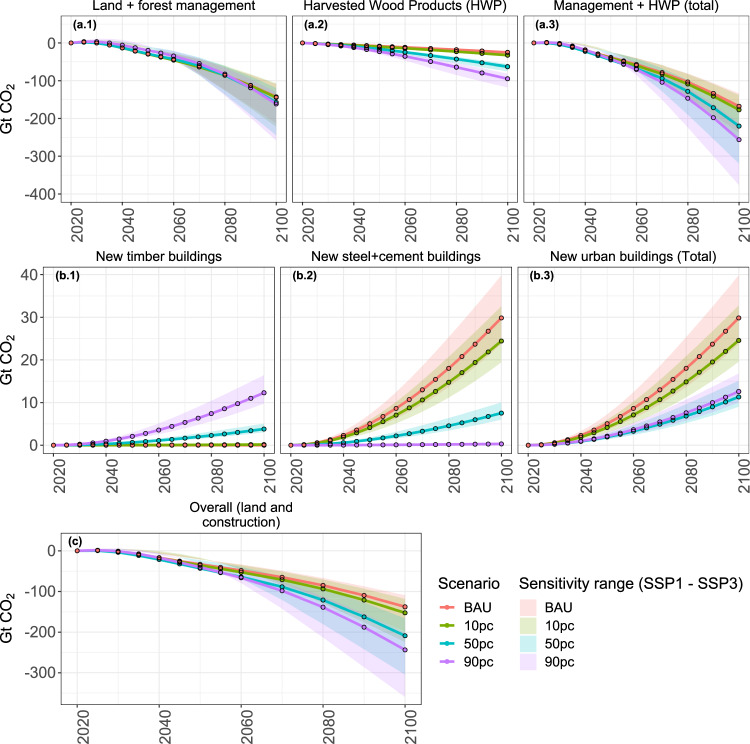
Comparison of global cumulative emissions from land use and construction material manufacturing by 2100 (with respect to 2020). Solid lines represent emissions in engineered wood demand scenarios in an SSP2 world. The transparent area shows the emission range between SSP1 and SSP3 scenario. **a.1** Emission from land and forest management (calculated as the sum of gross land-use change emissions and regrowth emissions from forests^11^, see Supplementary Fig. 9). **a.2** long-term carbon storage in harvested wood products (calculated as the sum of carbon storage in industrial roundwood and buildings made out of engineered wood^11^ Supplementary Fig. 10). **a.3** Land-use change emissions (sum of **a.1** and **a.2**^11^). **b.1** Emissions from the manufacturing of wood for new urban buildings. **b.2** Emissions from the manufacturing of conventional raw materials (cement and steel) for new urban buildings. **b.3** Emissions from manufacturing and processing of raw materials for construction of new urban buildings (sum of **b.1** and **b.2**). **c** Overall cumulative emissions from land and construction material processing (sum of **a.3** and **b.3**).

Our results highlight that ambitious engineered wood demand scenarios (50 pc and 90 pc) lead to lower land-related cumulative CO_2_ emissions than the BAU and 10 pc scenarios (Fig. 3a.3). In our baseline scenario (BAU), we observe cumulative land-related CO_2_ emissions of −168 Gt CO_2_ by the end of the century from land-use change (see “Methods” for components of land-use change emissions in MAgPIE). In higher engineered wood demand scenarios, the cumulative land-related emission savings, when compared to the BAU scenario range from 9 Gt CO_2_ (5% lower emissions compared to the BAU scenario, ca. 0.12 Gt CO_2_ yr^−1^) in the 10pc scenario, 53 Gt CO_2_ (31% lower emissions compared to the BAU scenario, ca. 0.66 Gt CO_2_ yr^−1^) in the 50 pc scenario and 89 Gt CO_2_ (53% lower emissions compared to the BAU scenario, ca. 1.11 Gt CO_2_ yr^−1^) in the 90pc scenario. Emission savings in the land-use sector are driven by higher regrowth (carbon uptake in both natural vegetation and plantations) (Fig. 3a.1 and Supplementary Fig. 9), and higher long-term carbon storage in timber buildings (Fig. 3a.2 and Supplementary Fig. 10). Even though gross land-use change emissions are higher in scenarios with high engineered wood demand, the accompanying carbon uptake by regrowth of forests more than compensates for those emissions (Supplementary Fig. 9).

Complementary to land-related CO_2_ emissions and carbon stored in harvested wood products, we calculate and compare CO_2_ emissions from the manufacturing and processing of engineered wood for construction (Fig. 3b.1) and conventional raw materials for construction (cement and steel) (Fig. 3b.2) to be used in new urban buildings, based on emissions factors from the literature^6^. Emissions related to the manufacturing of raw material for new urban buildings made from engineered wood are shown in Fig. 3b.1. By 2100, CO_2_ emissions in the 50pc and 90pc scenarios are 4 Gt CO_2_ and 12 Gt CO_2_, respectively (emissions in 10 pc and BAU scenario are negligible or zero). By including land-related emissions and tracking long-term carbon storage in HWP, our approach allows for a more accurate assessment of CO_2_ emissions and savings related to engineered wood production and use for the majority of HWP’s life cycle. Emissions related to the manufacturing of cement and steel for new urban buildings are shown in Fig. 3b.2. By 2100, CO_2_ emissions in the BAU and 10 pc scenario are 30 Gt CO_2_ and 24 Gt CO_2_, respectively, which is three to four times higher than the emissions in the 50 pc scenario (8 Gt CO_2_). Emissions in the 90pc scenario are negligible.

Overall emissions related to the manufacturing of raw materials for new urban buildings (consisting of both conventional and timber buildings) are shown in Fig. 3b.3. By 2100, CO_2_ emissions in the BAU scenario (30 Gt CO_2_) are about three times as high compared to the 50 pc (11 Gt CO_2_) or 90 pc (13 Gt CO_2_) scenario, while the emissions in 10 pc scenario (25 Gt CO_2_) are only slightly lower than in the BAU scenario. The 90 pc and 50 pc scenarios are not very different from each other with regard to cumulative emissions of 13 Gt CO_2_ and 11 Gt CO_2_, respectively, by 2100 (Fig. 3b.3).

Ambitious mitigation strategies using engineered wood as construction materials result in lower overall cumulative emissions (Fig. 3c). In our baseline scenario (BAU), we observe overall emissions of −138 Gt CO_2_ by the end of the century. In higher engineered wood demand scenarios, the overall emission savings, when compared to the BAU scenario, range from 14 Gt CO_2_ (10% lower emissions) in 10 pc scenario, 71 Gt CO_2_ (51% lower emissions) in 50pc scenario and 106 Gt CO_2_ (77% lower emissions) in 90 pc scenario. This is due to the synergistic effect of both lower net land-related emissions (Fig. 3a.3) and emissions avoided by moving away from conventional construction material manufacturing (Fig. 3b.3). The mitigation potential of using engineered wood for construction purposes between 2020 and 2100 is estimated to be 0.18 Gt CO_2_ yr^−1^ in the 10 pc scenario, 0.89 Gt CO_2_ yr^−1^ in the 50 pc scenario and 1.32 Gt CO_2_ yr^−1^ in the 90 pc scenario.

It is important to point out here that the emission factors used here for calculating emissions related to the manufacturing of raw materials for new urban buildings are static and do not factor in any reduction pathway for construction material (steel and concrete) used in conventional buildings (see “Methods”). This means that, possibly, over time the emission savings will be smaller than indicated by our results even though currently, there is no clear sign of a large-scale "greening” of the conventional steel and cement production^23^.

### Net carbon storage in wooden buildings

**Fig. 4:**
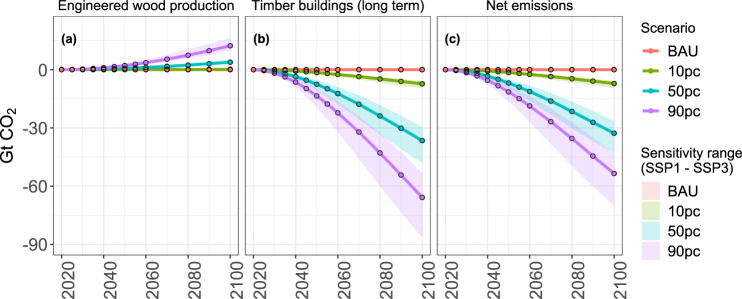
Comparison of global cumulative emissions associated with engineered wood usage in novel timber buildings until 2100 with respect to 2020. Solid lines represent emissions in engineered wood demand scenarios in an SSP2 world. The transparent area shows the emission range between SSP1 and SSP3 scenario. **a** Emissions from the production of engineered wood for a novel timber building. **b** Long-term carbon storage in novel timber buildings. **c** Net cumulative emissions associated with the construction of new urban buildings made of engineered wood (sum of **a** and **b**).

The amount of engineered wood used for constructing new urban buildings plays a major role in determining the amount of long-term carbon storage in the building sector. By the end of this century, the net carbon emissions from construction and usage of new urban buildings made of engineered timber are −7 Gt CO_2_ in the 10 pc scenario (0.3 Gt CO_2_ from the production of engineered wood and −7.3 Gt CO_2_ from long-term storage), −33 Gt CO_2_ in 50 pc scenario (4 Gt CO_2_ from the production of engineered wood and −37 Gt CO_2_ from long-term storage), and −53 Gt CO_2_ in 90 pc scenario (12 Gt CO_2_ from the production of engineered wood and −65 Gt CO_2_ from long-term storage). By the middle of this century, carbon stored in buildings made from wood (Fig. 4b and Supplementary Fig. 10b) will exceed the carbon stored in other wood products (i.e., industrial roundwood) (Supplementary Fig. 10a). In the long term, it can be seen that the long-term carbon storage potential in buildings made of engineered timber is many folds higher than the emissions associated with the production of engineered timber. The negative signs of the emissions presented here only reflect the carbon accounting logic in MAgPIE (see “Methods”) and do not correspond to the active sequestration of carbon from the atmosphere.

### Sensitivity analysis

To test the robustness of our results across different socioeconomic development pathways^1^, we perform a sensitivity analysis of our emission calculations with respect to SSP1^24^ and SSP3^25^ scenarios, which cover future socioeconomic development where challenges to adaptation and mitigation are both low (SSP1— sustainability) or both high (SSP3—regional rivalry)^1^. We choose the shared socioeconomic pathways SSP1 and SSP3 for the sensitivity analysis to cover a range of plausible assumptions for future socioeconomic development.

Both, cumulative emissions from land use (Fig. 3a.3) and overall cumulative emissions (Fig. 3c) are more sensitive to the socioeconomic developments in an SSP1 world than in an SSP3 world. For both land use and overall emissions, by 2100, we observe 42–48% lower cumulative emissions in an SSP1 world and 17–22% higher cumulative emissions in an SSP3 world when compared to our SSP2 results across all engineered wood demand scenarios discussed here. Cumulative emissions from new urban building construction (Fig. 3b.3) are also sensitive to SSP scenario assumptions with 31–36% higher emissions in an SSP1 world and 18–23% lower emissions in an SSP3 world by 2100. This behavior is driven by higher demand for construction material for new urban housing in a rapidly urbanizing world in SSP1 compared to either SSP2 or SSP3 scenarios. It can be inferred that the assumptions regarding socioeconomic development of the future have a relatively uniform impact on the cumulative emissions observed in all engineered wood demand scenarios.

Cumulative emissions linked to engineered wood usage in novel timber buildings (Fig. 4) are also more sensitive to the socioeconomic developments in an SSP1 world than in an SSP3 world. For emissions from the production of engineered wood, by 2100, we observe on average 25–33% higher cumulative emissions in an SSP1 world and 17–25% lower cumulative emissions in an SSP3 world when compared to our SSP2 results in the 50 pc and the 90 pc scenario (close to zero emissions from engineered wood production in the BAU and the 10 pc scenarios). Long-term carbon storage in timber buildings (Fig. 4b) is also sensitive to SSP scenario assumptions with 30–43% lower emissions in an SSP1 world and 14–20% higher emissions in an SSP3 world by 2100 across the engineered wood demand scenarios. Net cumulative emissions from the production and use of engineered wood for timber buildings is linked to 29–32% lower emissions in an SSP1 world and 14–19% higher emissions in an SSP3 world by 2100 across the engineered wood demand scenarios. Linked to the higher demand for construction material for new urban housing in a more urbanized SSP1 world, long-term carbon storage potential is higher when compared to both an SSP2 and SSP3 world. Even if such an urbanized world would end up emitting more CO_2_ during the production of engineered wood for novel urban housing of the future, the carbon storage potential more than compensates for these emissions in the long term.

## Discussion

We present a global estimate of land-use change and associated emissions (including carbon storage potential) associated with constructing mid-rise buildings made from engineered wood until 2100. We introduce a baseline (BAU) scenario where future urban building construction is based on conventional materials such as cement and concrete and, as a counterfactual, three scenarios where additional timber (as engineered wood) is demanded for construction purposes on top of regular timber demand. Our analysis compares the emissions from raw material production for conventional building materials and engineered wood, including competition for land and accounts for long-term carbon storage in future wooden buildings.

Our model produces reasonable global estimates for the observed data. In 2020, 132 Mha of plantation forest exists globally^21^ compared to our estimate of 138 Mha. The production volume coming from plantations in 2015 is likely more than 33%^9^ of the global production of industrial roundwood (1.8 billion m^3^) from all types of forests. This is comparable to the 34% contribution of plantations in overall industrial roundwood production from our estimates (Supplementary Fig. 18). We also show that when additional forest resources are used—the associated competition with agriculture for land might not be evident on a global or regional level but only on a finer spatial scale (Supplementary Figs. 8 and 13).

Engineered wood for construction purposes could also be an addition to other well-studied land-based mitigation options. Afforestation/reforestation, avoided deforestation, natural forest management, forest plantations, fire management, and avoided wood fuel harvesting are estimated to have a mitigation potential of 2.2 to 11.4 Gt CO_2_ yr^−1^^26–29^. Protecting, managing, and restoring forests and other ecosystems have a mitigation potential of 0.8 to 12.7 Gt CO_2_ yr^−1^^28^. Bioenergy with carbon capture and storage (BECCS) is estimated to have a mitigation potential of 0.5 to 11.3 Gt CO_2_ yr^−1^^26,28,30^. The mitigation potential of engineered wood as construction material discussed here (between 2020 and 2100, 0.18 Gt CO_2_ yr^−1^ in 10 pc, 0.89 Gt CO_2_ yr^−1^ in 50 pc and 1.32 Gt CO_2_ yr^−1^ in 90 pc scenario) is on the lower side of other land-based mitigation options. The mitigation potential of engineered wood as construction material estimated in this study only reflects emission savings from new construction of housing for novel urban dwellers. If existing buildings, after their serviceable or usable lifetime, would be partly replaced with wooden buildings, the mitigation potential of using engineered wood for construction would likely be higher. Moreover, we see most of the reduced carbon emission benefits only after mid-century (Fig. 3c), and some long-term carbon storage in HWPs would likely continue after 2100. Therefore, our annual mitigation estimates are rather conservative.

Buildings made from timber act as a long-term carbon sink of harvested wood^5,6^. Engineered wood used for the construction of buildings can substitute conventional, hard-to-decarbonize building materials^4–6^. This helps to avoid considerable CO_2_ emissions from the manufacturing of cement and steel (depending on the scenario). In addition, producing timber for buildings made from wood results in higher forest regrowth over time due to the establishment of new forest plantations on otherwise less productive land as well as a reduced share of production being sourced from natural vegetation, resulting in net carbon uptake rather than release. However, the increasing risk of forest disturbances under climate change^31^ with a negative impact on natural forest carbon stocks^32^, as well as plantation productivity and wood quality, could affect the regrowth potential. In the absence of catastrophic disturbances, higher CO_2_ levels, longer growing seasons and warmer temperatures might also be beneficial^33^ for forest growth and productivity in temperate and boreal forests^34^.

Unless large quantities of existing harvests are redirected to construction material production, meeting engineered wood demand for new urban housing would need additional harvests from forests. Given the global efforts to safeguard and protect existing pristine primary forests as well as secondary forests of high conservation value, the production of timber from such natural forests needs to be minimized or avoided completely. In the scenarios discussed here, protected areas (frontier forests, biodiversity hotspots, and land earmarked for protection by the International Union for Conservation of Nature (IUCN) category I and II, also see methods and Supplementary Fig. 12) are explicitly prohibited from timber production. Such land protection policies reduce the available options for harvesting natural forests. To compensate for restrictions in biomass removal from natural forests, a higher amount of timber production can come from highly managed forest plantations. However, higher harvest from forest plantations is also associated with declining biodiversity (measured in MAgPIE as Biodiversity Intactness Index (BII)) (Supplementary Fig. 11).

Our research also has some caveats. All scenarios discussed here do not account for the impacts of future climate change on agriculture and forestry. We also do not account for future biogeochemical changes (CO_2_ fertilization, temperature and precipitation changes, disturbance regimes, etc.) that come with future climate change. It has been estimated^35^ that forest output could increase by ~30% over the century under high levels of warming (9 W m^−2^), and by about 11% under a mitigation scenario (3.7 W m^−2^) because of CO_2_ fertilization. Considering increased productivity under higher CO_2_ and the projected increased frequency and severity of disturbances leading to additional forest damage in an integrated way is, therefore, a key future research challenge to better understand global forest health and carbon uptake.

Currently, most of the wood needed for construction is softwood because of material characteristics but also because most of the wood processing machinery used for making construction-grade wood is also adapted to process softwood rather than hardwood. In the future, under the engineered wood demand scenarios discussed here, we assume that hardwood also (in conjugation with softwood) can be used for engineered wood production^6,36^. Timber processors would need to invest in and acquire machinery that can handle hardwood, entailing a major transformation of the wood-producing industry, which is still centered around softwood. There is also evidence that hardwood is potentially equally suited for construction purposes^37–40^ if the industry and regulatory frameworks adapt to a changing supply. With MAgPIE, we focus on an amalgamated assessment of the land-use impacts and mitigation potential of engineered wood as construction material at a global level rather than a detailed analysis of the wood sector. This is one of the reasons for not differentiating between softwood and hardwood in this analysis, complementing the reasons related to industry and technology.

Forest growth curves also dictate the relationship between time and estimated carbon sequestration in trees. Net carbon emissions in the first cycle of newly established forest plantations depend on up-front emissions from land conversions and subsequent carbon sequestration modeled via changes in age-class structure—a dynamic that is likely sensitive to the choice of growth curves. A flatter growth curve at the beginning of forest growth would result in a longer time needed to capture back the carbon emissions from the first cycle of forest plantation establishment. Alternatively, the realization of a steeper growth curve in a forest would likely result in earlier recouping of carbon emissions from the first cycle of newly established forest plantations. In this study, we did not perform a sensitivity analysis of the changes in growth curve assumptions made in MAgPIE.

Our results can be interpreted as a lower bound for future engineered wood demand for construction as we only account for newly built housing in developing regions. We disregard the potential replacement or sustainable renovation of depreciated buildings in developed regions like Europe and North America, where urbanization levels are expected to stay stable over time^1^. This is an oversimplification as even though construction activities would go on with a stable urban population—our scenarios simplify the engineered wood demand calculations in this regard. Our study design further assumes little migration from rural to urban areas and minimal demand for engineered wood in developed regions. This would likely change if developed world regions include future urban construction with engineered wood in their sustainability goals. Nevertheless, this study highlights the importance and climate benefits of creating a long-term carbon pool in buildings made from engineered wood.

Increased demand for engineered wood would also require active reforestation, as higher demand for engineered wood (on top of normal demand for roundwood) means that the trees should be planted at an increasing pace now for meeting the engineered wood demands of the future. At the same time, when competing with other land resources, converting land into forests increases the solar radiation absorbed in such converted land and reduces the amount of light and heat that can be reflected into space. Therefore, despite their carbon sequestration benefits, land converted to forests via afforestation or otherwise, may exert a net warming influence via the albedo effect, especially in boreal regions^41^. We did not account for the impact of such an albedo effect in this study.

Increased forest harvesting would need to be ensured as part of an overall commitment to sustainable forest management and governance. One way to make sure that the sourcing of engineered wood meets fair standards, would be to improve and promote stringent and verifiable forest certification schemes (e.g., Forest Stewardship Council) and strengthen existing global forest governance initiatives and policies (e.g., https://www.euflegt.efi.int to combat illegal logging) and support capacity building to strengthen forest governance.

Future construction of buildings with engineered wood is usually touted as a novel climate change mitigation option. It could reduce GHG emissions^42^ from the building sector while reducing the costs related to overall construction^43^. Use of engineered wood in buildings is already associated with fire^44^ and earthquake resistance^45^, lower construction times^46^ and reduced waste^46^ during construction. The building sector offers a unique opportunity for decarbonization. Substituting a major portion of raw material needed for residential building construction for new urban population with engineered wood provides a lucrative option for long-term carbon storage in buildings.

Increased demand for engineered wood results in lower overall emissions via the construction of new urban buildings, while additionally offering a long-term carbon sink to mitigate climate change. It does so, however, at the risk of depleting existing natural carbon sinks through the removal of biomass. The long-term carbon storage potential in timber buildings, while explicitly accounting for competition for limited land resources between alternative land uses, i.e., agriculture and forestry, has not yet been quantified. We find that sourcing of engineered wood (even in the high-end demand scenarios discussed here) does not result in much higher CO_2_ emissions from deforestation as compared to a business-as-usual scenario (Fig. 3a.1).

We show that if 90% of the new urban population would be housed in newly built urban mid-rise buildings with wooden constructions, 106 Gt additional CO_2_ (Fig. 3g) could be saved by 2100 which is about 10% of the remaining carbon budget for the 2 ^∘^C climate guard rail. Wood plantations would need to expand by an additional 143 Mha in this scenario compared to a scenario where such construction with wood does not exist. Understanding the land-use implications of increased engineered wood demand for construction provides a unique perspective into a highly relevant climate change mitigation option. Forest land’s competition for limited land resources under additional engineered wood demand scenarios does not come at the cost of agricultural land. A doubling of land-use intensification between 2020 and 2100 to increase the productivity of agricultural land would be strong enough to meet production requirements from agricultural land in all scenarios (Supplementary Fig. 8). Production of engineered wood for building purposes is within the forest use constraints posed by ambitious biodiversity conservation scenarios.

The Paris Agreement and recommendations by the Intergovernmental Panel on Climate Change (IPCC) state that limiting human-induced global warming to a specific level would require limiting cumulative anthropogenic CO_2_ emissions^47^. This creates an imperative to study long-term carbon storage in timber buildings as a mitigation option. The land-use modeling framework introduced here can help to provide a more consistent basis for tracking land use on a global and regional scale for assessing land-use change impacts and associated GHG emissions from global forest change under increased demand for engineered wood.

This study indicates that the transition to the deployment of large-scale urban housing made from engineered wood can be a viable mitigation option to combat climate change. Under strict forest and biodiversity protection scenarios, which are effective in conserving forest cover and forest carbon stocks compared to unprotected areas^48^, high demand for engineered wood will likely result in the loss of unprotected areas as well as its biodiversity (Supplementary Fig. 11). The loss of such unprotected ecosystems should not be downplayed as they could have a key role in global carbon cycle variations^49^. Currently, unprotected areas may need additional protection, e.g., if it harbors endemic species, to avoid negative spillover effects on biodiversity from the interplay of forest and biodiversity protection, wood production, and agricultural expansion. Even though the land expansion needed for forest plantation in the highly engineered wood demand scenarios is unlikely to benefit biodiversity (Supplementary Fig. 11), it has fewer effects than a BECCS scenario^50,51^ or expansion of conventional agriculture^52^. This paper helps to include these issues to a certain extent by excluding protected areas from being used for timber production, which helps toward achieving the life-on-land sustainable development goal (SDG 15).

We also show that while the results of this analysis depend on the choice of socioeconomic development assumptions for the future, the overall trend is robust. Even though our results are robust, the wide range of possible future socioeconomic developments in different SSPs brings a wide range of uncertainty about the overall cumulative emissions from both land-use and construction of new urban buildings. Uncertainties in future socioeconomic developments also include dietary patterns. Transition to timber cities would synergize closely with living in a sustainable world with healthy diets from sustainable food systems. Transition to healthy diets is shown^53^ to free-up land resources, making additional land available for the establishment of forest plantations and afforestation, reducing the need to clear natural forests further.

Progress in both forest management and the construction sector could increase the supply as well as the demand for engineered wood. Practical forest management and timber processing innovations will likely emerge from a transition to timber cities. Satisfying increased timber demand would also need gradual ramping up of engineered wood production capabilities. By extension, expansion of forest plantations would be needed, with strong natural forest and other vegetated land protection policies.

No existing research to our knowledge quantifies the optimal forest area needed for transition to timber cities while accounting for competition with agriculture on a finer spatial scale. Current understanding of this transition to timber cities is either limited in geographical scope^54–57^ or ignores competition between agriculture and forest-land use^6,54,58,59^. Existing studies on timber cities of the future also assume that standing forests can be harvested based on the growing stock potential of forests^59^. These assumptions ignore the biodiversity impacts of increases in harvest levels. These studies also assume that if forests can be managed in a way that the harvesting rate is no more than additional increments, then, the overall forest management is sustainable, neglecting effects on other ecosystem services and biodiversity. These existing research gaps are addressed directly by our study. Our land-use modeling framework can be directly used by other studies at global and regional levels, but additional analyses would be required for designing more accurate future engineered wood demand scenarios. Current Integrated Assessment Models and other global land-use models used to calculate the global carbon budgets and land-use emissions should account for this mitigation option and study its uncertainties for a better understanding of this additional long-term carbon storage pool in the form of harvested wood products.

## Methods

### Land-use model

MAgPIE is a global multi-regional land-system modeling framework^19,20,60^. It is a global partial equilibrium model that minimizes production costs while producing food, feed, bioenergy, and timber throughout the 21st century. MAgPIE is programmed to run recursive-dynamically with limited foresight. The mathematical programming model is written in GAMS and solved with CONOPT4 solver^19^. MAgPIE chooses optimal land-use patterns, yields, and total costs of agricultural and roundwood production for every simulation unit, called cluster^19^. MAgPIE’s clusters are spatial units (aggregated from data on 0.5° resolution)^19,61^. Spatially explicit biophysical data derived from the Lund-Potsdam-Jena managed Land (LPJmL) model^62^ which acts as an additional constraint within MAgPIE. The demand for agricultural commodities (food, feed, etc.) is calculated based on population and income projections for the 21st century from the Shared Socioeconomic Pathway 2 (SSP2) scenario^1^. The demand for timber (industrial roundwood, wood fuel, and wood for building material) is driven by urban population change in an SSP2 world while also accounting for stringent land protection measures.

### Forest-land dynamics

Forest-land dynamics are modeled via two separate but synergistic representations in MAgPIE (Forestry and Natural Vegetation)^11^. The forestry representation in MAgPIE defines highly managed plantations with age-class dynamics, afforestation, and above-ground carbon dynamics. The forestry sector also deals with afforestation for Carbon dioxide removal (CDR) and timber production. Afforestation, according to existing National Policies Implemented (NPI) until 2030, supporting the Paris Agreement, is modeled by default as regrowth of natural vegetation^63^ where the regrowth of natural vegetation follows S-shaped growth curves which are parametrized based on^64^. New forest plantations are established in the simulation step to account for existing timber demand. The rotation length in forest plantations is governed by the maximization of the current annual increment^11^. The forest plantation area is initialized based on the Forest Resource Assessment (FRA) national-level data on planted forests^21,65,66^. During the land-use optimization stage of our model, a forest plantation can be converted to cropland. Forest plantations, once established, cannot be converted to another land-use type until the time equivalent to the prescribed rotation lengths has elapsed^11^. The forest plantation areas which are mature can be harvested and converted to cropland if need be. MAgPIE further captures indirect land-use changes where forest plantations established on cropland can lead to the displacement of cropland to other areas (e.g., m natural forests) causing deforestation.

The natural vegetation representation includes primary forests, secondary forests, and non-forest natural vegetation (termed other land in MAgPIE). The representation accounts for land and carbon stock dynamics, modeled endogenously for primary forests (forests with no visible sign of human intervention), secondary forests (forests with some indication of human intervention and management) and other land (degraded forests or uncultivated land with lower vegetation carbon density than normal forests (<20 tC/ha))^11^. The initial spatial distribution of the sub-land-types of primary forest, secondary forest, and other natural land is based on the land use Harmonization (LUH) dataset^67^. The area allocated to primary forests is assumed to exist in the highest age class in 1995 and secondary forests follow an age-class structure during the first simulation period^68^. In MAgPIE, projected biomass availability from forests does not account for future pests and disease prevalence or storm damage as they are not explicitly modeled by LPJmL, which is providing the underlying vegetation carbon simulations. We also do not account for changes in forest growth, forest composition, and biomass availability due to forest fires and heat stress.

MAgPIE simulates two different kinds of land protection, i.e., (a) logging restriction due to protection based on the World Database on Protected Areas (WDPA) maintained by the International Union for Conservation of Nature (IUCN) along with complete avoidance of timber logging in Frontier Forests (FF) and Biodiversity Hotspots (BH), and (b) land protection based on NPIs and nationally determined contributions (NDC) to the Paris Agreement. Land protection based on WDPA, FF, and BH (collectively referred to as protected area(s) in the manuscript) is achieved by 2030. NPI/NDC policies ramp up until 2030 and are assumed constant thereafter. For WDPA, the level of land protection is based on IUCN category I+II, which reflects areas currently under protection (e.g., strict nature reserves and national parks), and is distributed equally across all sub-land-types (primary forest, secondary forest, and other natural land). Both wood and wood fuel can be produced from primary and secondary forests, but other land is only allowed to be harvested for wood fuel. After the initialization/base year, the development of forest cover and different types of forest areas is modeled endogenously and depends on the demand for forest products, harvest costs, allowable harvest volumes per area, demand for other land uses, land-use change costs, and other land-use change constraints.

### Timber demand

In MAgPIE, three categories of wood products—industrial roundwood, wood fuel, and engineered wood can be harvested from forests (primary forest, secondary forest, and forest plantations). Demand for industrial roundwood and wood fuel is calculated based on current demand for these products, population, and income changes^11^. Engineered wood demand for construction is derived based on the amount of peak population expected to live in cities in an SSP2 world. Engineered wood demand is calculated as a function of the new influx in urban population and woody biomass demand per capita^6^.

1 $${M}_{2020}^{{t}^{*}}=({P}_{{t}^{*}}-{P}_{2020})\cdot {M}_{w}^{c}\cdot CW\cdot PR$$

In Eq. (1), ${M}_{2020}^{{t}^{*}}$ is the country-level cumulative demand for engineered wood after 2020 until urban population peaks in time t^*^ for each country. ${P}_{{t}^{*}}-{P}_{2020}$ is the difference in urban population between 2020 and peak urban population in time t^*^. ${M}_{w}^{c}$ is the mean woody biomass demand for primary and structural systems in the buildings. CW is the carbon-to-wood ratio and is set to be the global average of 0.476 ± 0.04 gC/gDM. PR is the scenario-specific factor for demand in timber cities and amounts to 0.1, 0.5, and 0.9 for 10 pc, 50 pc, and 90 pc scenarios, respectively. Cumulative demand from equation (1) is converted to annual demand^18^. Transition to timber cities is assumed to be a gradual process and for this reason, cumulative to annual demand conversion is based on a simple sliding scale where higher weight is given to the demand closer to the time of peak urban population and a harvesting efficiency of 50% is assumed for engineered wood production.

For this analysis, industrial roundwood and wood fuel demand are the same across all the scenarios mentioned above. Only engineered wood demand differs among scenarios following Eq. (1). This helps to isolate the direct impact of only engineered wood demand on land-use change and associated emissions. There is always a temporal lag between demand and supply for wood. New forest plantations are established based on the currently existing roundwood demand. These forest plantations are protected for the full duration of the prescribed rotation length. For example, if the prescribed rotation length at a given location is 40 years, then the forest plantation is established in the year 2060 based on the demand in the year 2100, and they would only be harvested in the year 2100. Assumptions regarding the choice of rotations lengths (along with general harvesting decisions to meet roundwood demand) are based on assumptions made for forest sector implementation in MAgPIE^11^.

### Trade

MAgPIE simulates agricultural^19,69^ and timber trade^11^ among world regions ensuring that the regional demand for food, feed, and timber can always be met by domestic production and imports from other regions. The global trade balance in MAgPIE ensures that the global production stays larger than or equal to the global demand^69^. In MAgPIE, agricultural and timber products demand in a region can be fulfilled in two ways: (1) self-sufficiency pool based on historically observed region-specific trade patterns, and (2) comparative advantage pool based on optimal cost-efficient production.

In the self-sufficiency trade, regional self-sufficiency ratios define the demand in each world region (for traded goods, i.e., agricultural and timber) to be fulfilled by domestic production. Self-sufficiency ratios smaller than one indicate that the region imports from the world market, while self-sufficiency greater than one indicates that the region produces for export^69,70^. MAgPIE also accounts for trade costs (including trade margins and trade tariffs) to decide optimal production strategies. The share of regional demand to be fulfilled via the self-sufficiency pool is determined by a trade balance reduction factor for each commodity^69^. When trade balance reduction is 1, all demand is fulfilled via the self-sufficiency pool.

In the comparative advantage pool, global supply is assumed to be larger or equal to demand. The model can allocate production freely across the globe based on comparative advantages. When the trade balance reduction factor equals 0, all demand is fulfilled via the comparative advantage pool. As the transition to timber cities discussed here can be seen as an indication of a world moving towards sustainable living, we use a globalized trade specification in MAgPIE for trade to be closer to globalization assumptions from such a world^70–73^.

### Land-use change emissions

Net CO_2_ fluxes from land use, land-use change, and forestry (LULUCF) i.e., land-use change emission in MAgPIE include CO_2_ fluxes due to biomass removals (for roundwood production including engineered wood), deforestation (i.e., clearing forest for alternative land use, not including forest-type conversion), afforestation, shifting cultivation (deforestation followed by abandoning) and regrowth of forests following biomass removal or abandonment. Burning wood fuel after harvest and conversion of forests to agricultural land leads to the release of CO_2_ into the atmosphere. Afforestation, regrowth, and long-term carbon stored in harvested wood products lead to CO_2_ sinks.

In MAgPIE, we account for (1) gross land-use change emissions (i.e., land-use change emissions not including regrowth), (2) emissions due to natural forest degradation (as part of gross land-use change emissions) based on historically observed rates^74^, (3) regrowth in forests and other land, (4) long-term carbon storage in wood products, and (4) slow release of CO_2_ into the atmosphere from industrial roundwood and engineered wood due to decay (with an assumed half-life of 35 and 60 years, respectively) based on tier 1 methodology of The Intergovernmental Panel on Climate Change (IPCC)^75^.

In MAgPIE, the carbon accounting logic for land-use change emissions accounts for gross land-use change emissions (i.e., land-use change emissions not including regrowth) with a positive sign; regrowth in forests and other land with a negative sign; as well as long-term carbon storage in wood products with a negative sign (as this pool is recalculated from emissions already assumed to have been lost to the atmosphere through roundwood harvesting). We also calculate the slow release of CO_2_ back into the atmosphere from these wood products due to decay with a positive sign.

In MAgPIE, we account for CO_2_ emissions from land clearing for plantation establishment. Therefore, the subsequent carbon uptake in plantations modeled via changes in age-class structure is implicitly diminished by the up-front emissions for land clearing in the first cycle. Depending on which type of land was converted to a plantation, this might result in net positive CO_2_ emissions in the first cycle of a plantation (for instance, if the natural forest was cleared for plantations establishment). Changes in the age-class distribution in forest plantations (and natural forests) and calculation of rotation lengths are based on the forest plantation growth curves assumptions^64^ and age-class structure^68^ used in MAgPIE^11^.

### Emissions from the manufacturing of raw materials for new urban buildings

Emissions from the manufacturing of raw materials for new urban buildings are based on the mean floor area per capita for residential buildings (m^2^/capita), material intensities for primary structural systems and enclosure systems (kg/capita) and emission factors associated with the production of construction materials (tCO_2_e/t)^6,76^. The emission factors include emissions from the site of raw material extraction, transportation to manufacturing facilities, and material manufacturing. We use the latest available emission factors for concrete^76^ and assume these emission factors to be static and do not factor in any CO_2_e reduction pathway for the production cycle of these materials. With a fundamental transformation toward renewable energy sources and the decarbonization of the global energy grid, the overall emission factors of these materials will be reduced. This has not been accounted for in this study.

### Carbon storage in harvested wood products

Several studies have reported on the benefits of using woody biomass on local, regional, and global scale for construction purposes^6,54–59,77,78^. Those studies usually focus on the role of engineered wood—a type of engineered wood product with a large surface area, like thick wood panels (natural or glued, or laminated). One such category of engineered wood is cross-laminated timber (CLT), which is a multi-layered wood panel product using at least three layers of parallel boards glued together with adhesive^44^. The perpendicular arrangement of panels in CLT provides a high level of stability, strength, and stiffness. Using engineered wood in mid-rise buildings has been recently touted as a potential replacement for traditional building materials in primary structure and enclosure^6^.

In MAgPIE, long-term carbon storage in harvested wood products (HWPs) is calculated^79^ based on IPCC tier 1 guideline^75^ while also calculating for the slow release of CO_2_ from existing wood product pool into the atmosphere from decay. Relevant equations dealing with long-term carbon storage potential in engineered wood are shown in Eq. (2). Carbon stored in HWPs (including engineered wood) affects national GHG inventories, where the production and end-use of HWPs play a key role^80^.

2a $${C}_{{{{{{{{\rm{t}}}}}}}}+1}={e}^{-{{{{{{{\rm{k}}}}}}}}}\cdot {C}_{{{{{{{{\rm{t}}}}}}}}}+\left(\frac{1-{e}^{-{{{{{{{\rm{k}}}}}}}}}}{k}\right)\cdot inflo{w}_{{{{{{{{\rm{t}}}}}}}}}$$

2b $${{\Delta }}{C}_{{{{{{{{\rm{t}}}}}}}}}={C}_{{{{{{{{\rm{t}}}}}}}}+1}-{C}_{{{{{{{{\rm{t}}}}}}}}}$$

Here, *C* is the carbon stock in buildings made from wood at the beginning of year *t* in Mt C. This value is zero until 2020, i.e., the point until which we assume no considerable carbon stock exists in buildings made from timber globally. *k* is the decay constant of first-order decomposition for engineered wood yr^−1^. *k* takes a value of ln(2) half-life^−1^ where half-life of engineered wood is assumed to be 60 years. inflow is the inflow to the non-decayed engineered wood pool during year *t* in Mt C yr^−1^. Δ*C* is the change in carbon stock in the engineered wood pool during year *t* in Mt C yr^−1^. Carbon stored in buildings is directly influenced by changes in the urban population.

To avoid double accounting of carbon sequestration in trees as well as long-term carbon storage in HWPs, we assume that whenever roundwood (industrial roundwood or wood fuel) is produced, all the carbon stored (via sequestration) in roundwood is lost to the atmosphere. Then a three-step process is implemented to balance carbon accounting from land-use change^11^: (1) all the carbon stored in harvested industrial roundwood is removed from our land-use change emissions, (2) we assume that all the wood fuel produced from harvesting would be lost to the atmosphere before the next simulation period, (3) we update stock of carbon stored in HWPs (industrial roundwood) along with the slow release of carbon from decomposition happening in the existing stock of HWPs. Decomposition emissions are then added back to land-use change emissions.

Even though we do not explicitly calculate end-of-life wood disposal, we account for the release of carbon from HWPs due to decay in our calculations. This also makes sure that the storage in HWPs (including engineered wood used in construction) is not permanent. The carbon storage pool in HWPs is regularly updated based on recurrent carbon outflows by applying a first-order decay function with constant annual decay factors. The outflows are counted as positive emissions released into the atmosphere. The methodology followed for this calculation is based on 2013 Revised Supplementary Methods, and Good Practice Guidance Arising from the Kyoto Protocol^75,81^ and is a part of Eq. (2).

### Reporting summary

Further information on research design is available in the Nature Research Reporting Summary linked to this article.

## Supplementary information

Supplementary information

Reporting Summary

## Data Availability

The processed model input data generated in this study have been deposited in the Zenodo database^82^. The numerical scenario results and source data are provided with this paper (including data presented in the supplementary information) and also hosted on Zenodo^82^. Instructions for reproduction and the analysis scripts supporting the findings of this study are available at Zenodo^83^.

## Code availability

The source code for MAgPIE version used in this study (v4.3.5) is publicly available at https://github.com/magpiemodeland Zenodo^84^. The model documentation is available at https://rse.pik-potsdam.de/doc/magpie/4.3.5/.
