# Supplementary material for: Land use change and carbon emissions of a transformation to timber cities
Source: Nat Commun. 2022 Aug 30;13:4889. doi: 10.1038/s41467-022-32244-w (PMC9427734; doi:10.1038/s41467-022-32244-w)
Supplement: Supplementary file 1 — Supplementary information [file 41467_2022_32244_MOESM1_ESM.pdf]

Supplementary information to the manuscript: Land use change and carbon emissions of a transformation to timber cities

Abhijeet Mishra[1,2,*], Florian Humpenöder[1], Galina Churkina[1], Christopher P.O. Reyer[1], Felicitas Beier[1,2], Benjamin Leon Bodirsky[1], Hans Joachim Schellnhuber[1], Hermann Lotze-Campen[1,2], and Alexander Popp[1]

[1]Potsdam Institute for Climate Impact Research (PIK), Member of Leibniz Association, P.O.Box 60 12 03, 14412, Potsdam, Germany
[2]Humboldt University of Berlin, Department of Agricultural Economics, Unter den Linden 6, 10099 Berlin, Germany
[*]mishra@pik-potsdam.de

Supplementary information

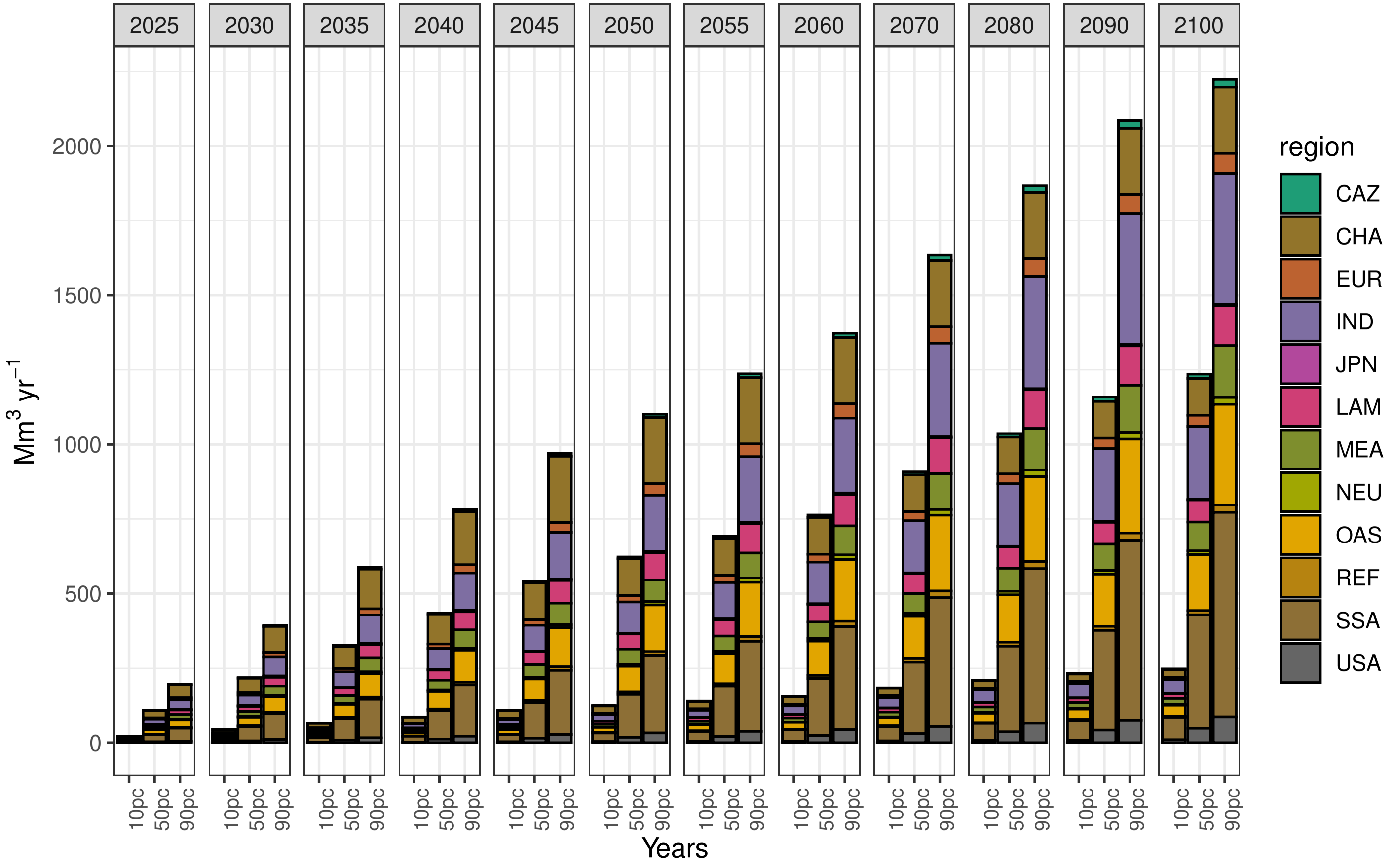

Supplementary Figure 1: Estimated engineered wood demand between 2025-2100 for construction of new urban buildings in MAgPIE world regions in the SSP2 scenario.

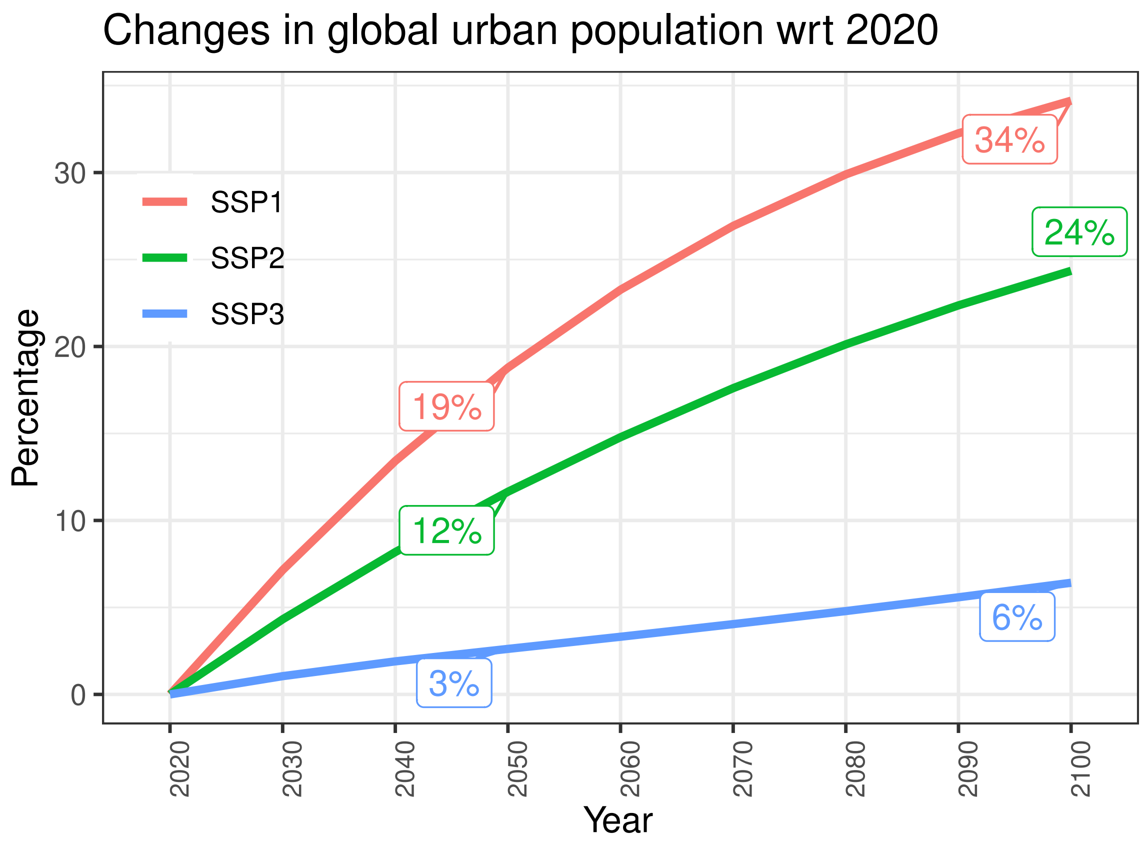

Supplementary Figure 2: Changes in the global urban population by 2100 with respect to 2020 in SSP1, SSP2 and SSP3 scenarios (values in SSP scenario specific colored boxes are shown for 2050 and 2100).

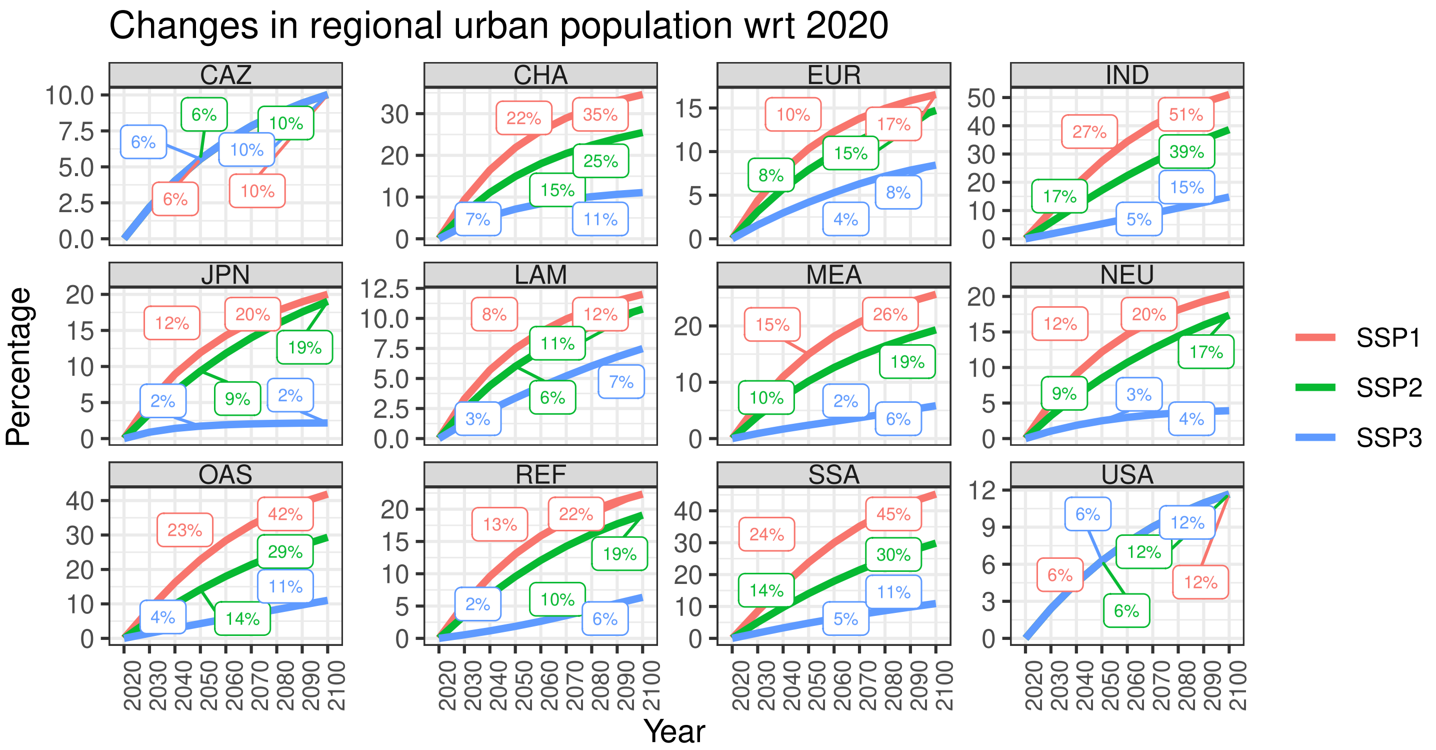

Supplementary Figure 3: Changes in the regional urban population by 2100 with respect to 2020 in SSP1, SSP2 and SSP3 scenarios (values in SSP scenario specific colored boxes are shown for 2050 and 2100).

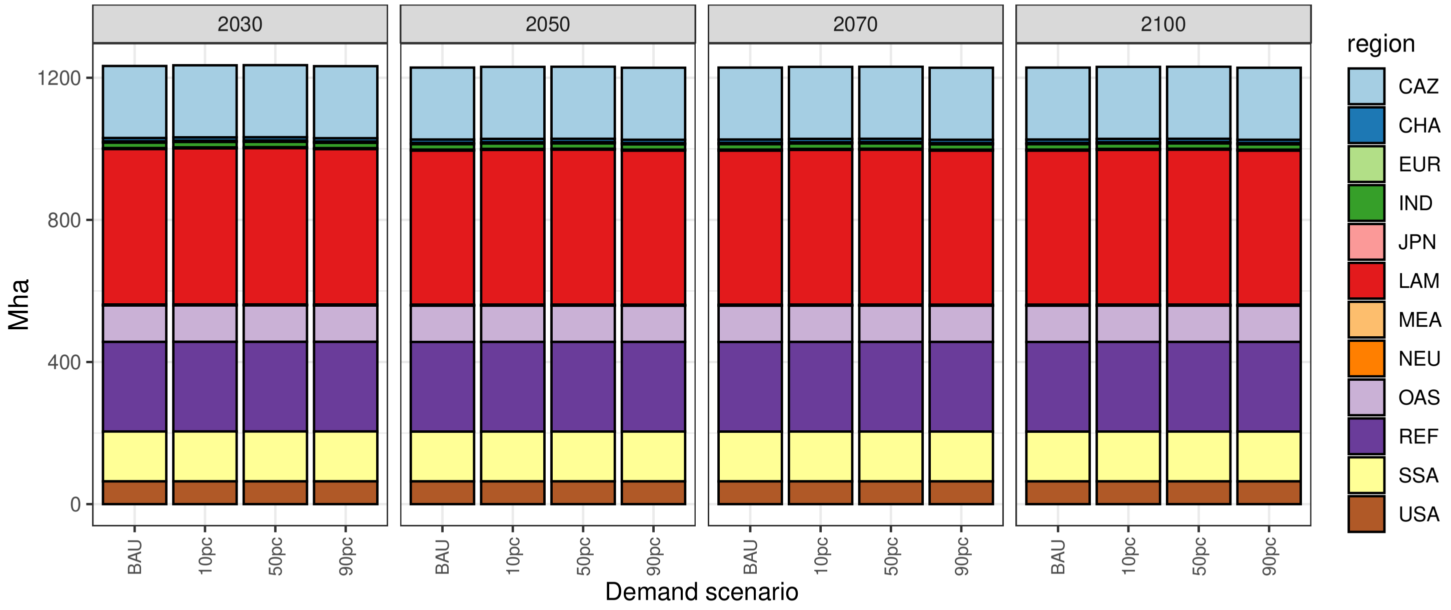

Supplementary Figure 4: Regional primary forest area evolution in the year 2030, 2050, 2070 and 2100 for all engineered wood demand scenarios in an SSP2 world. Changes in primary forest area between engineered wood demand scenarios are negligible as almost all primary forest is protected under FFBH protection scenario after 2030.

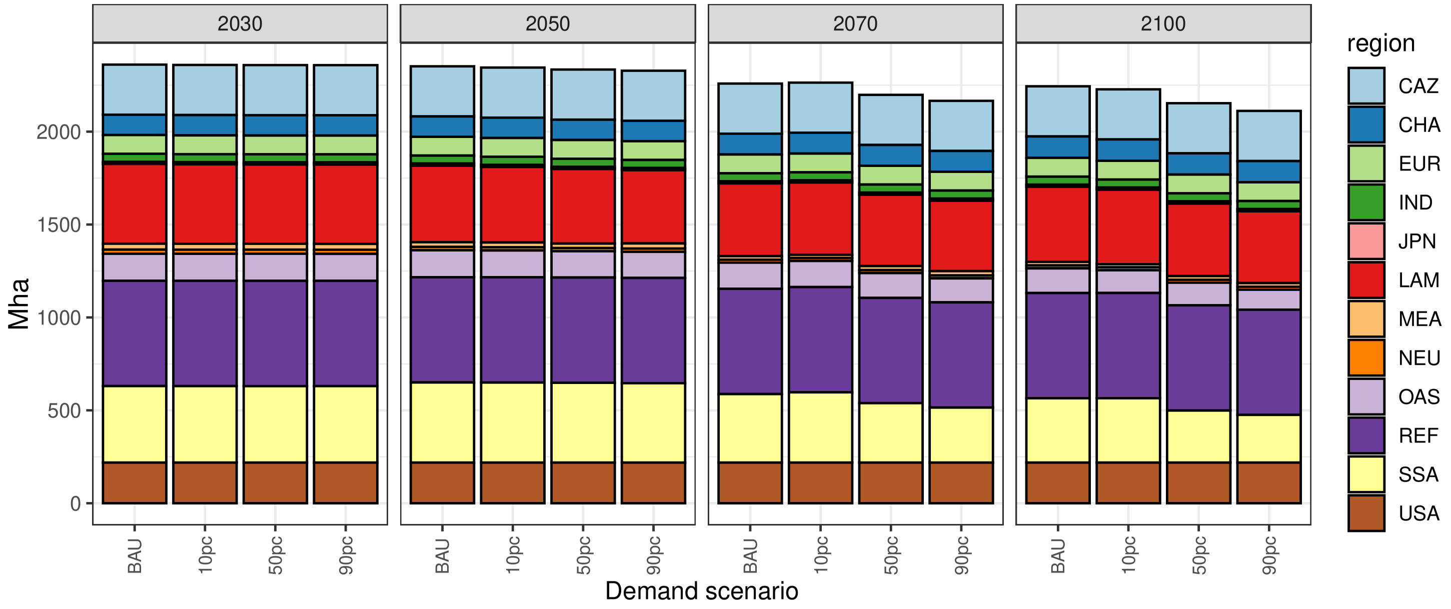

Supplementary Figure 5: Regional secondary forest area evolution in the year 2030, 2050, 2070 and 2100 for engineered wood demand scenarios in an SSP2 world.

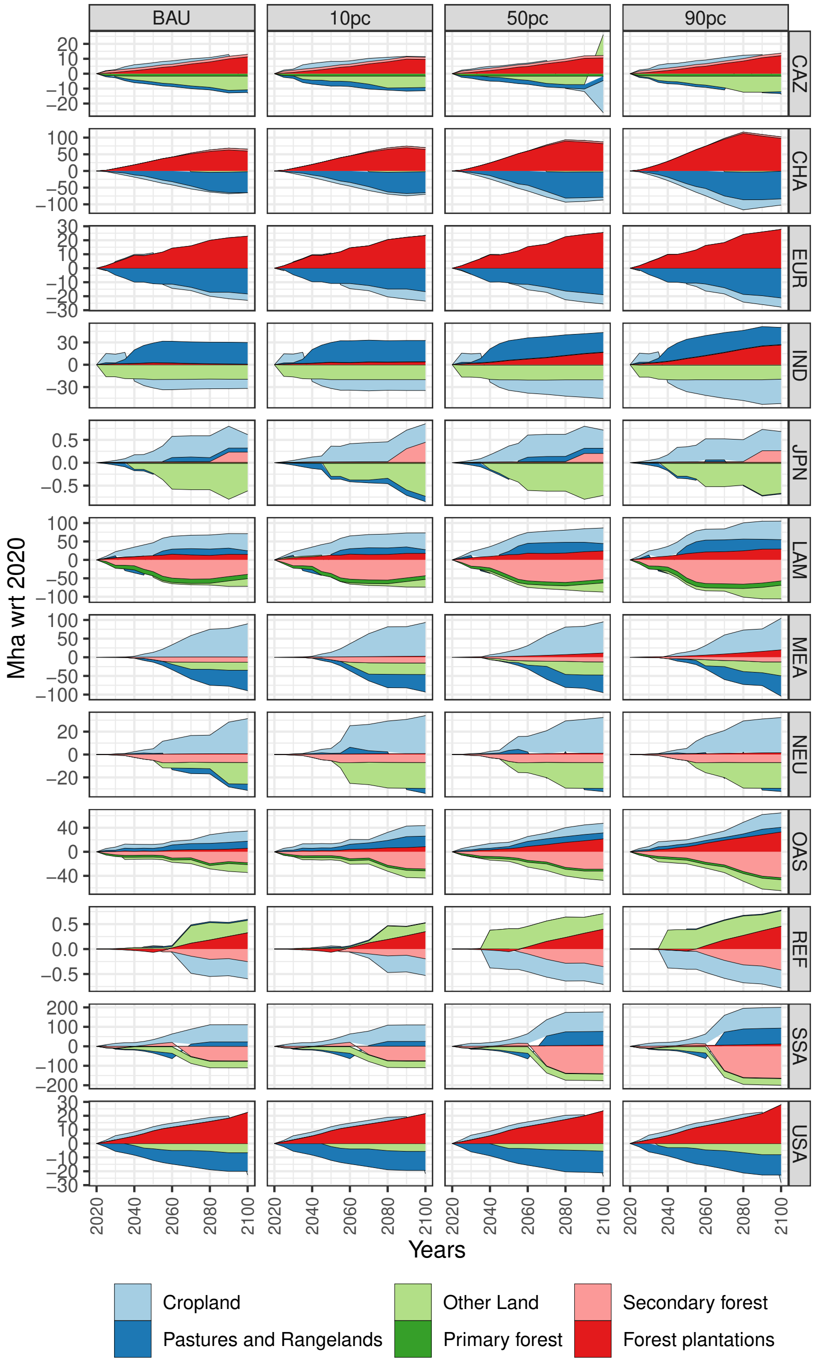

Supplementary Figure 6: Regional land use change between 2020-2100 with respect to 2020 (in Mha) in an SSP2 world for cropland, pasture and rangeland, natural vegetation (other land in MAgPIE), primary forests, secondary forests, and forest plantations. Values above 0 indicate increase in land-use compared to 2020 and values below 0 indicate decrease in land-use compared to 2020 for individual land-use types.

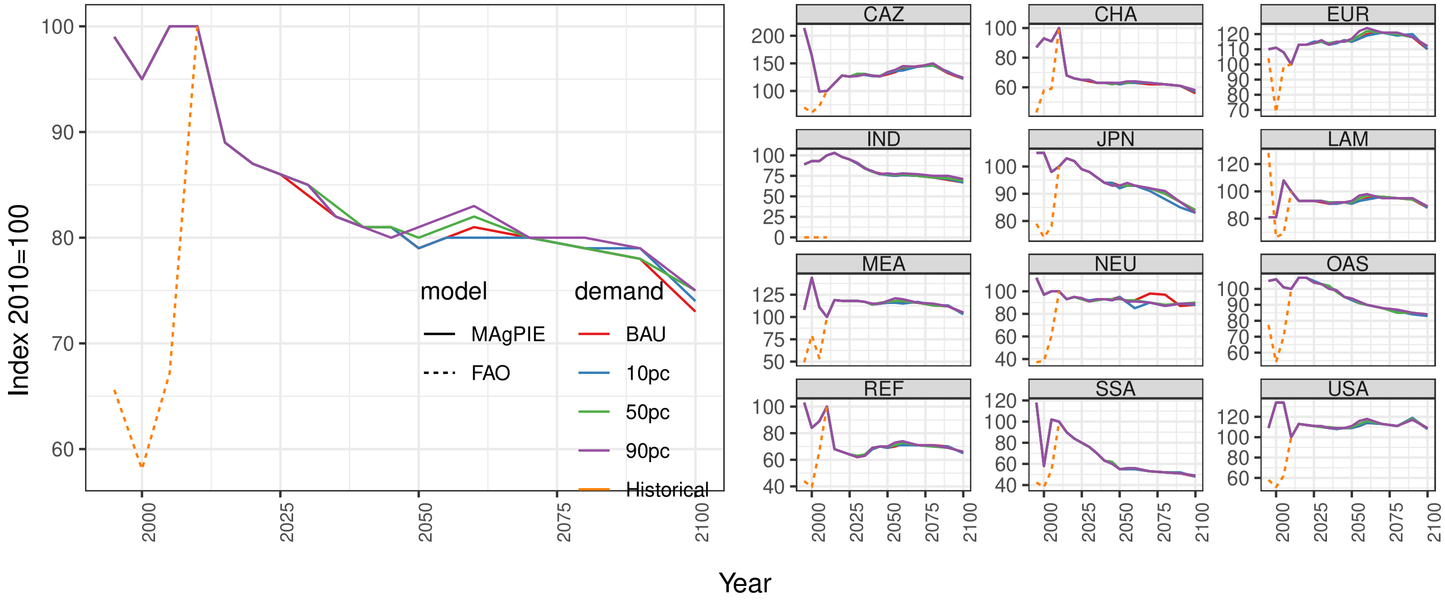

Supplementary Figure 7: Global (left panel) and regional (right panels) estimated agricultural commodity price index for period from 1995 to 2100 in an SSP2 world. 2010 is the base year (Index = 100). The price index is calculated based on the agricultural consumption basket of the base year (2010) and valued at consumer prices as reported by MAgPIE. This calculation is based on Laspeyres price index calculation method.

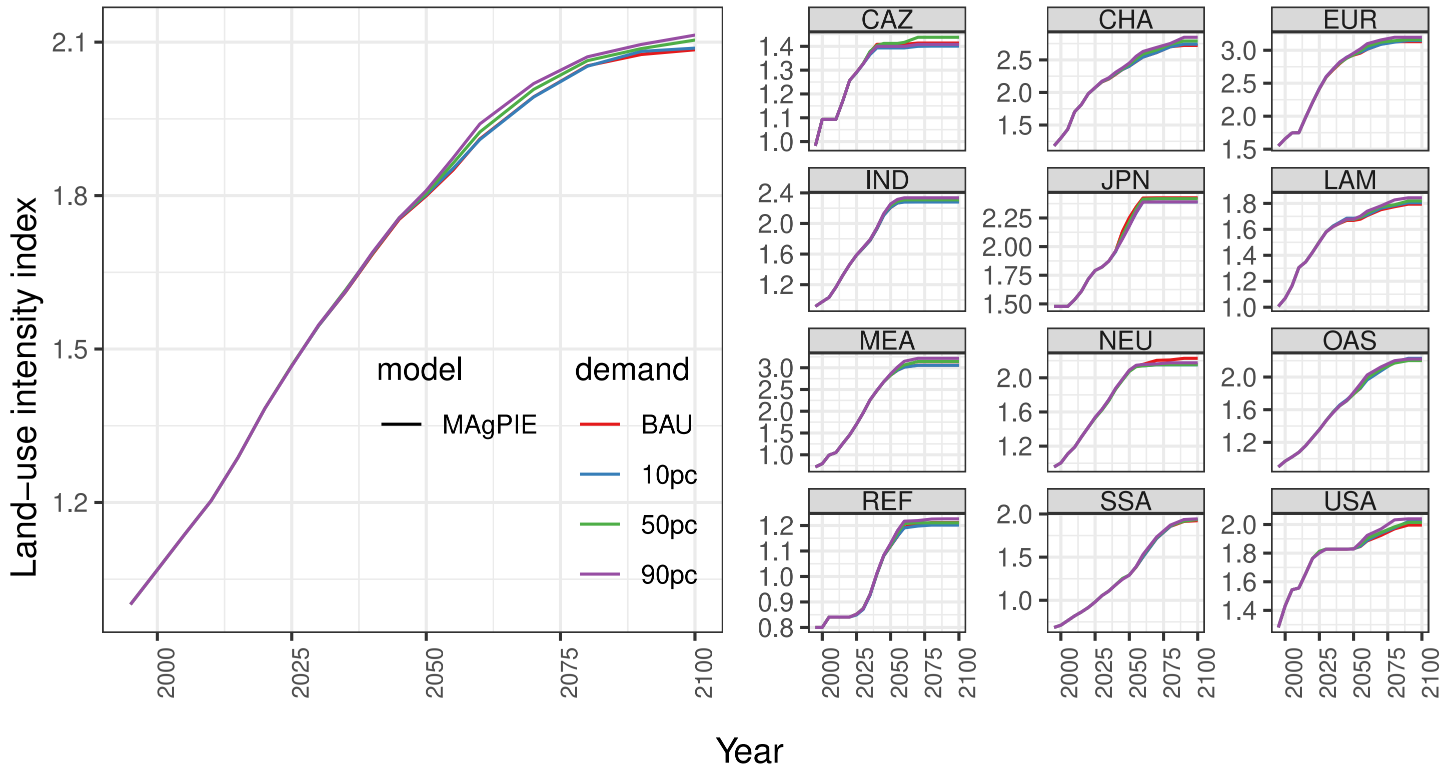

Supplementary Figure 8: Global (left panel) and regional (right panels) estimated land use intensity indicator (τ)[1] between 1995-2100 in an SSP2 world. τ in MAgPIE is a surrogate measure representing land-use intensity. Relative changes in τ are directly proportional to relative changes in agricultural land-use intensity. For example, τ doubles if crop yield doubles owing to improved management, technological development, or any other human activity.

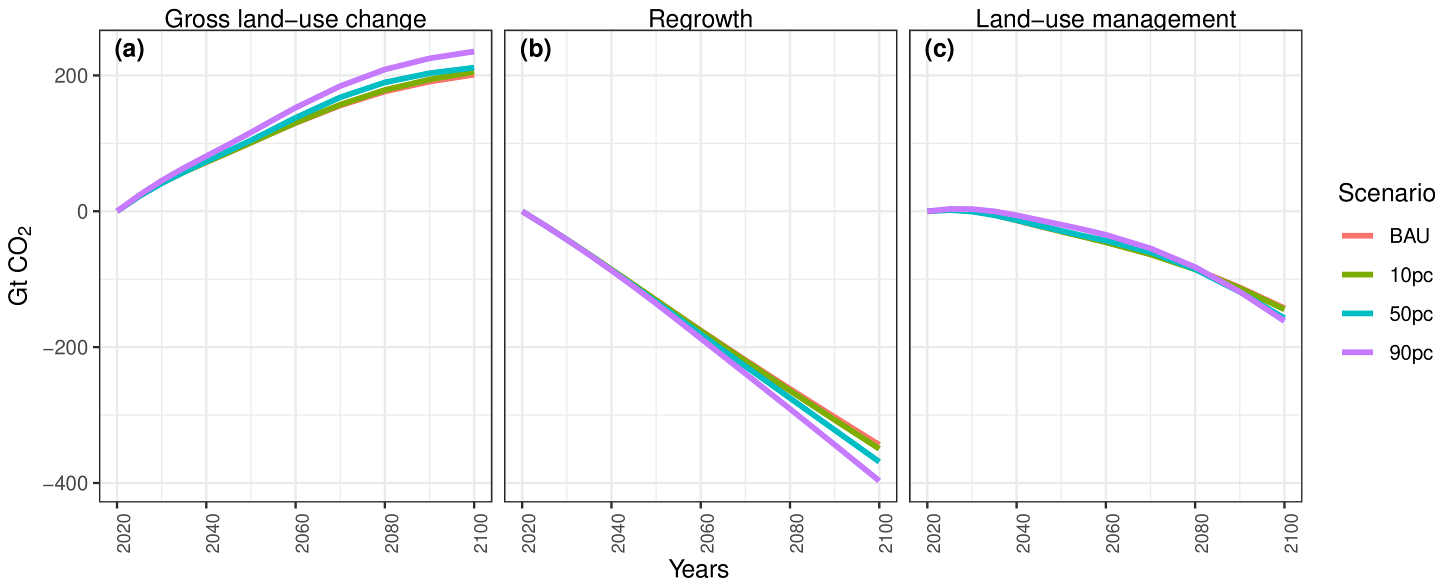

Supplementary Figure 9: Global cumulative emissions in an SSP2 world from land use management by 2100 (with respect to 2020). (a) Positive gross land use change emissions, (b) negative regrowth emissions from carbon uptake in forests and natural vegetation and (c) land use change management emissions (sum of panel (a) and (b)).

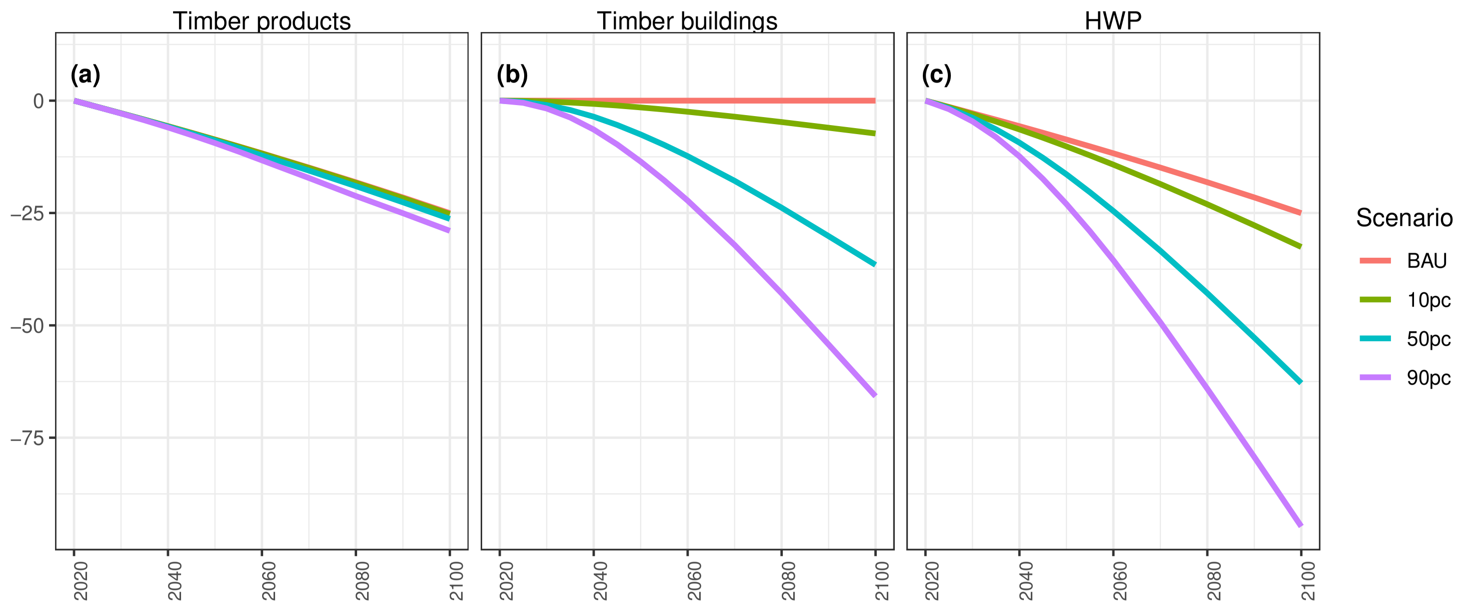

Supplementary Figure 10: Global cumulative emissions stored in harvested wood products in an SSP2 world until 2100 (with respect to 2020). (a) long-term carbon storage in harvested industrial roundwood, (b) long-term carbon storage in new urban buildings made out of wood, and (c) long-term carbon storage in harvested wood products (sum of panel (a) and (b)).

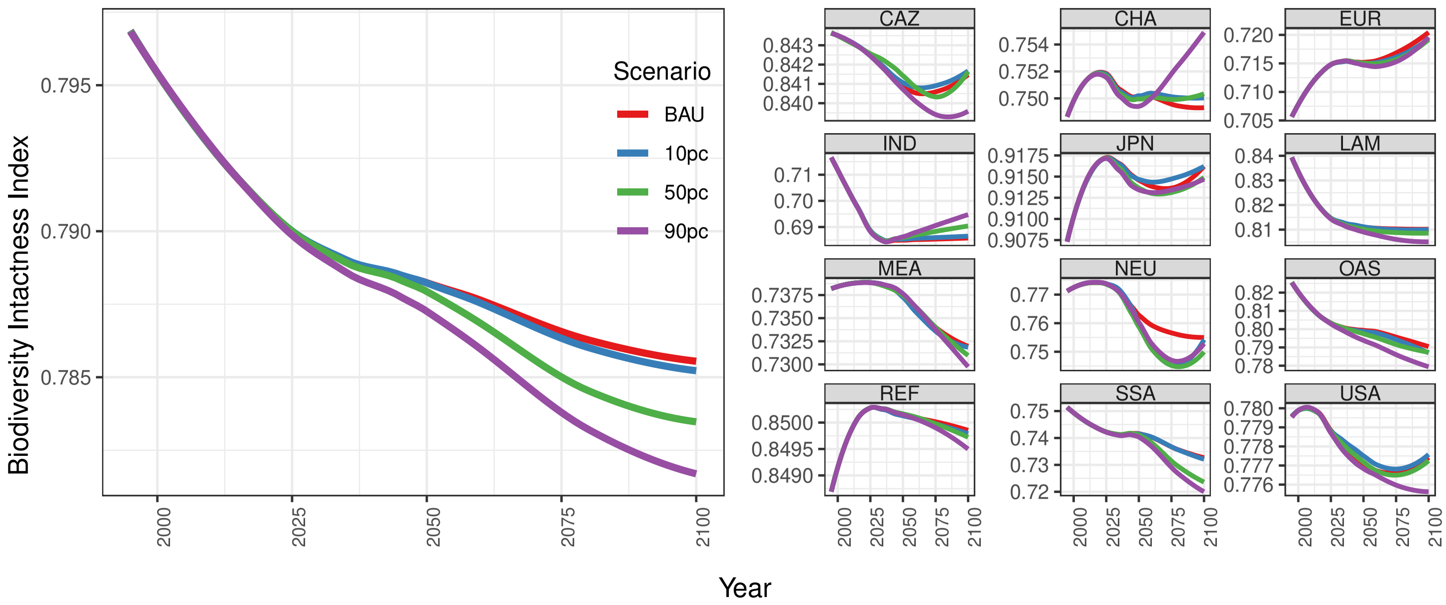

Supplementary Figure 11: Global (left panel) and regional (right panels) Biodiversity Intactness Index (BII) between 1995-2100 in an SSP2 world for different engineered wood demand scenarios in MAgPIE.

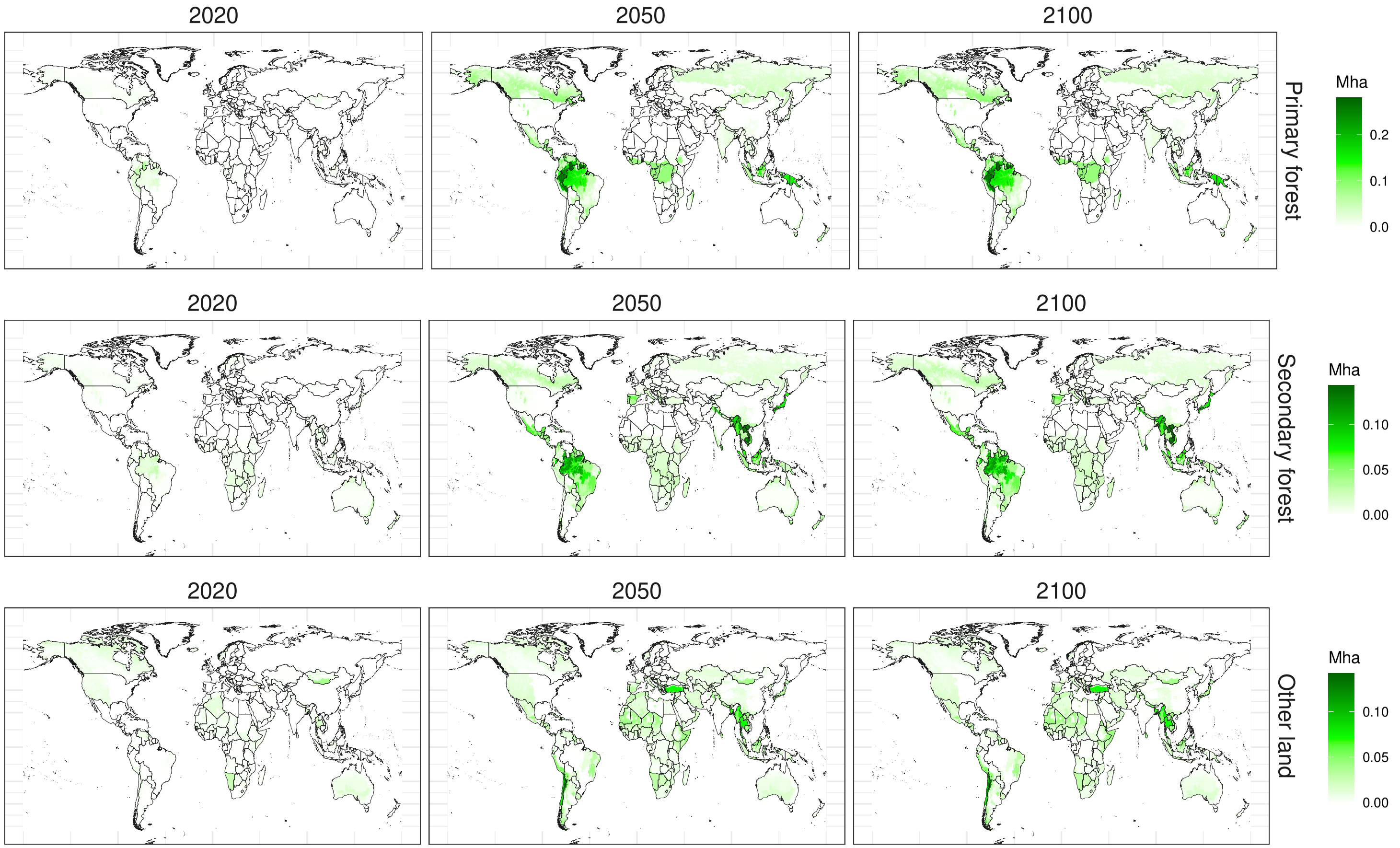

Supplementary Figure 12: Protected area in Mha including frontier forests, biodiversity hotspots and IUCN designated areas for different forest categories (primary forest, secondary forest and other land) over time.

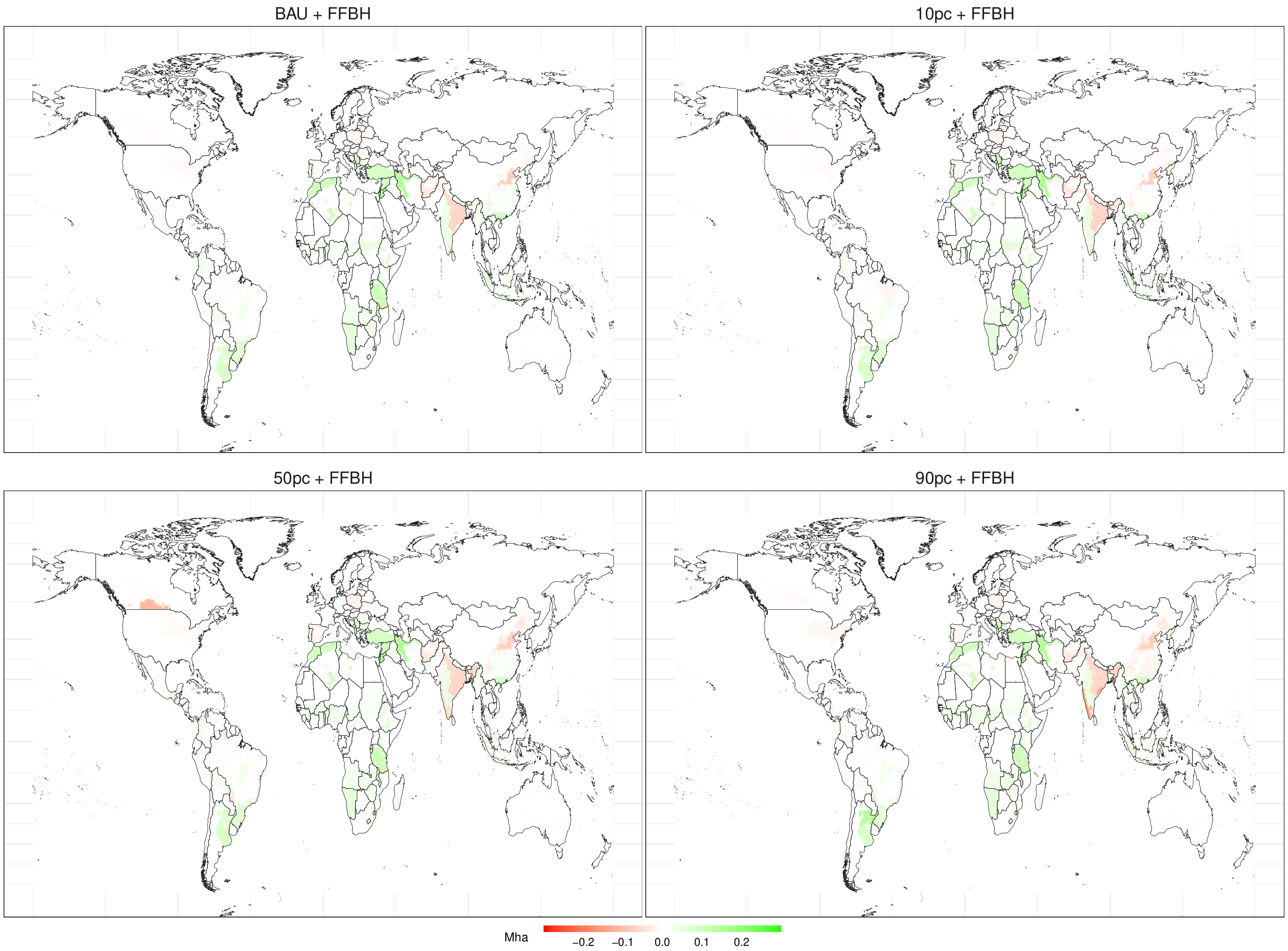

Supplementary Figure 13: Cropland difference in Mha between 2100 and 2020 in an SSP2 world. Shades of green represent increase in cropland area in 2100 compared to 2020 and shades of red represent decrease in cropland area in 2100 compared to 2020.

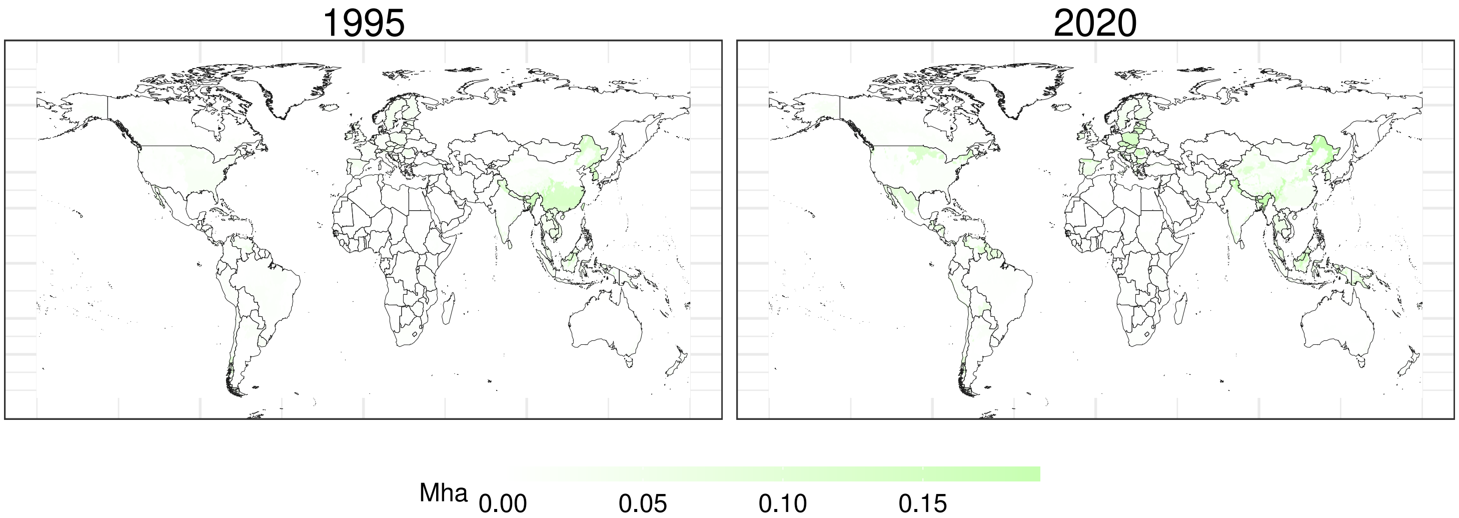

Supplementary Figure 14: Modeled forest plantation area in Mha in 1995 and 2020 in an SSP2 world. Forest plantations areas are initializated based on FRA 2015 data[2]

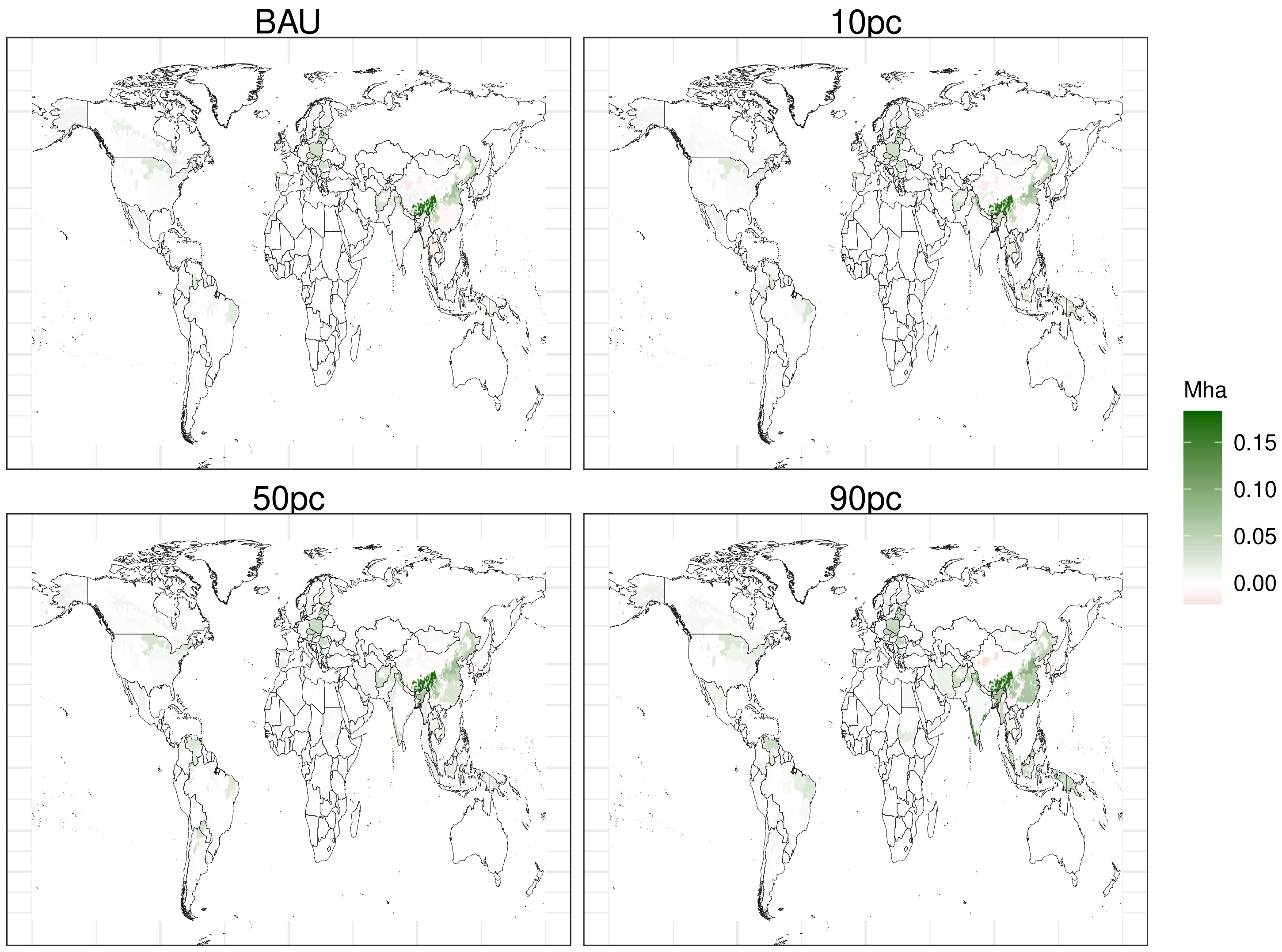

Supplementary Figure 15: Forest plantation area difference in Mha between 2020-2100 in an SSP2 world. Shades of green represent increase in forest plantation area in 2100 compared to 2020. Shades of red represent increase in cropland area in 2100 compared to 2020.

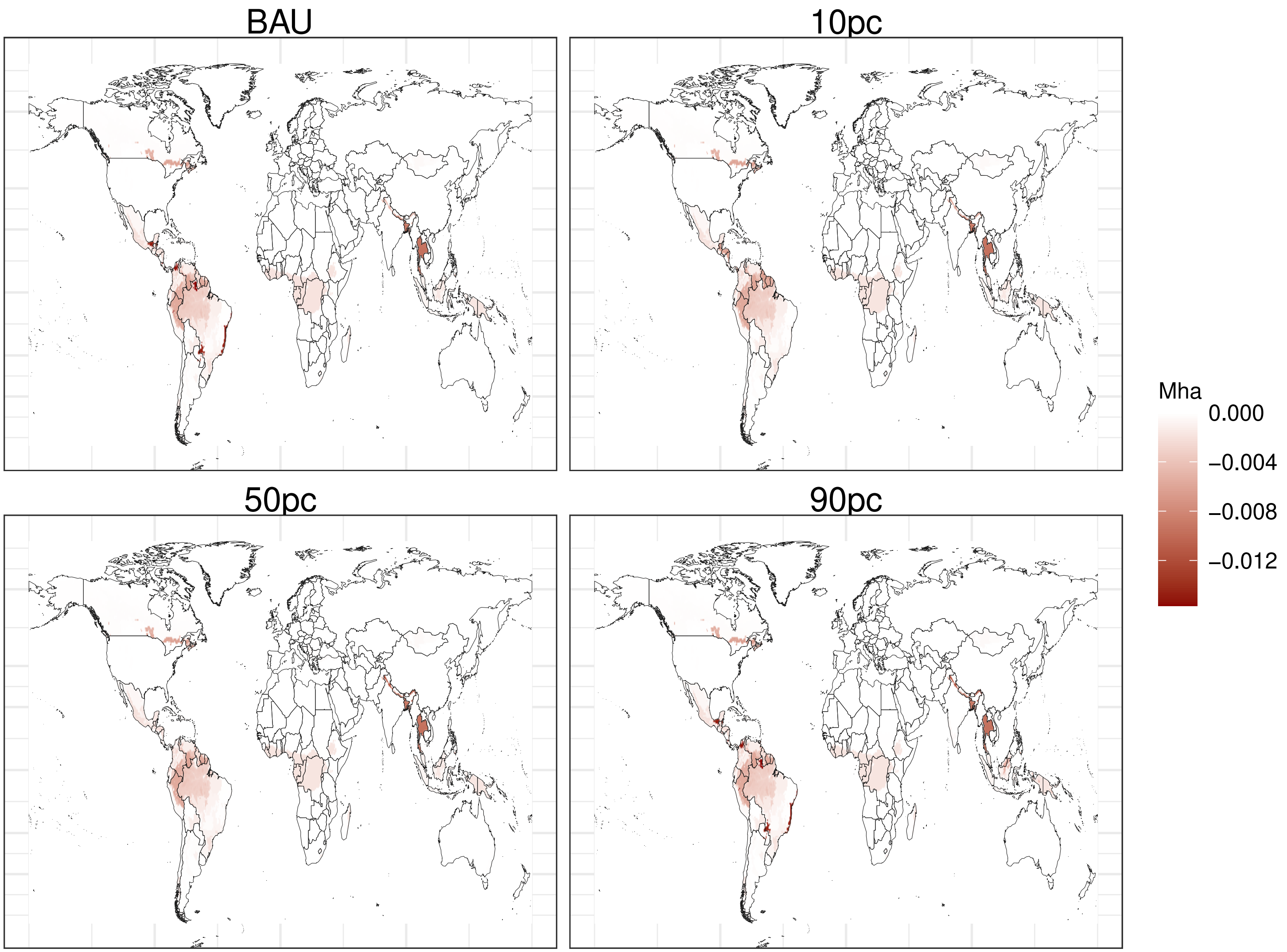

Supplementary Figure 16: Primary forest area difference in Mha between 2020-2100 in an SSP2 world. Shades of green represent increase in cropland area in 2100 compared to 2020. Shades of red represent increase in cropland area in 2100 compared to 2020.

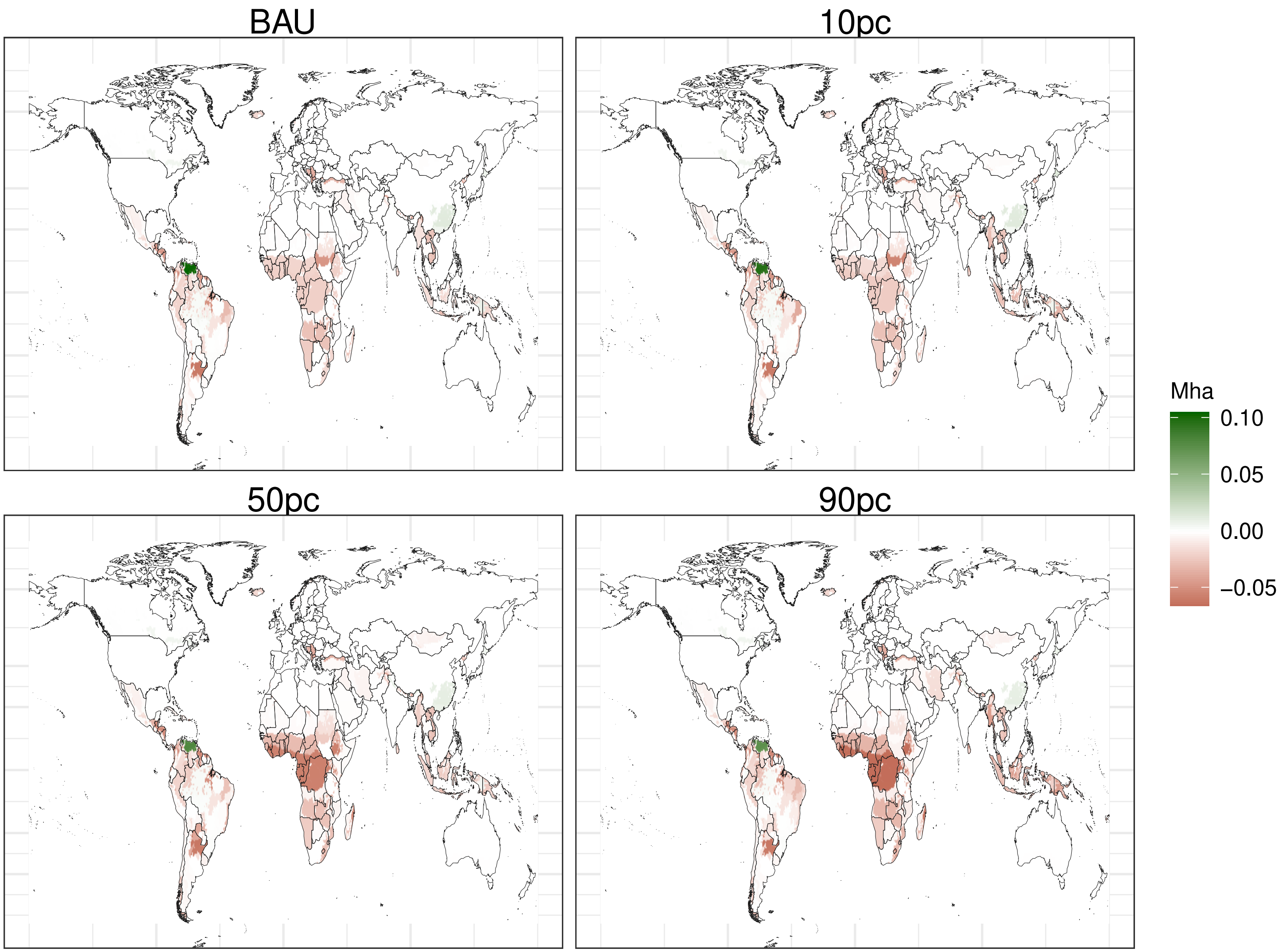

Supplementary Figure 17: Secondary forest area difference in Mha between 2020-2100 in an SSP2 world. Shades of green represent increase in cropland area in 2100 compared to 2020. Shades of red represent increase in cropland area in 2100 compared to 2020.

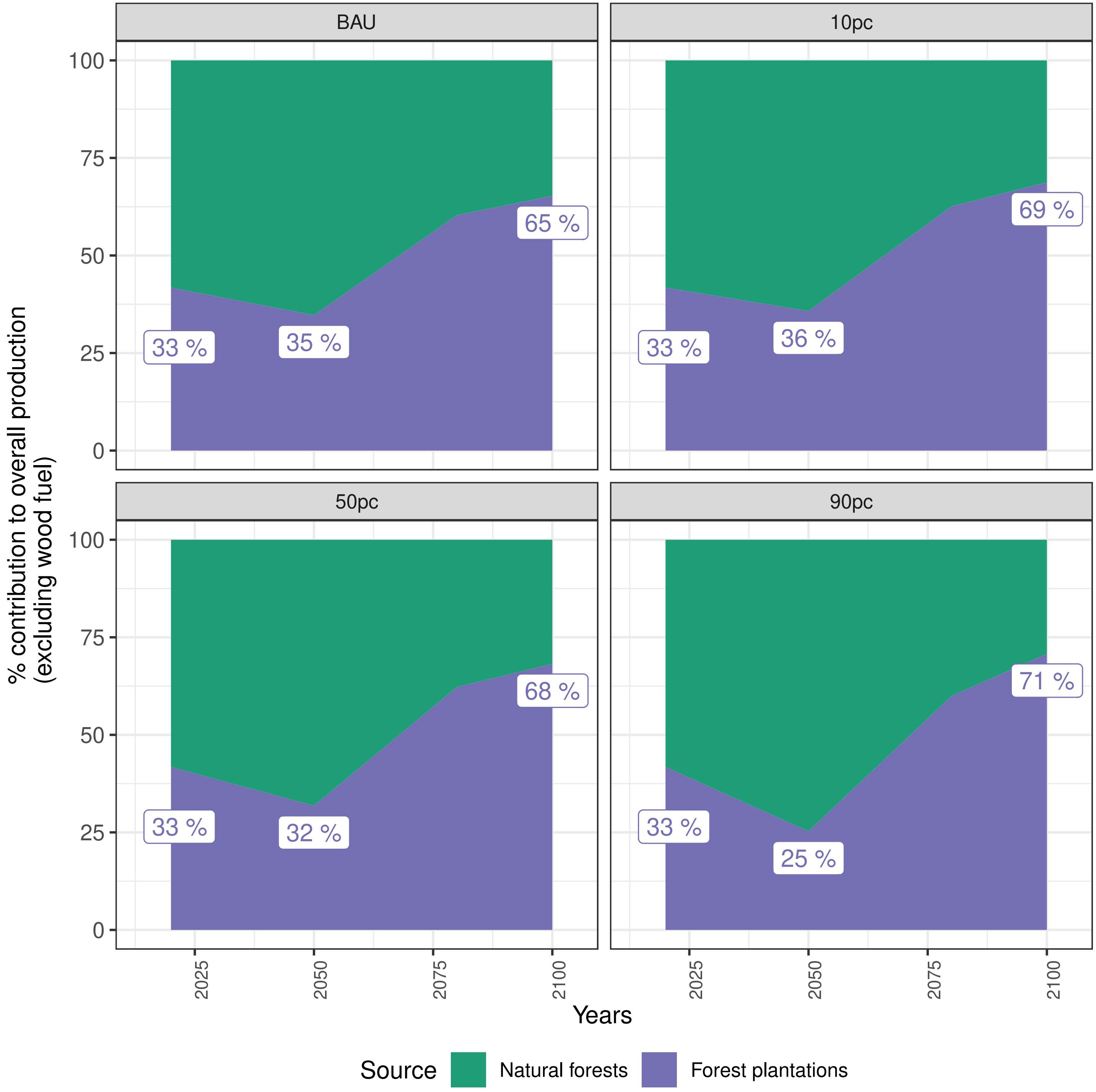

Supplementary Figure 18: Contribution of forest plantations (and natural forests) to global timber demand (including industrial roundwood and engineered wood, excluding wood fuel) in an SSP2 world.

Supplementary Table 1: ISO3 codes of countries belonging to standard MAgPIE regions.

Region name	MAgPIE Regions	ISO3 country codes
Canada, Australia, New Zealand	CAZ	AUS; CAN; HMD; NZL; SPM
China	CHA	CHN; HKG; MAC; TWN
Europe	EUR	ALA; AUT; BEL; BGR; CYP; CZE; DEU; DNK; ESP; EST; FIN; FRA; FRO; GBR; GGY; GIB; GRC; HRV; HUN; IMN; IRL; ITA; JEY; LTU; LUX; LVA; MLT; NLD; POL; PRT; ROU; SVK; SVN; SWE
India	IND	IND
Japan	JPN	JPN
Latin America	LAM	ABW; AIA; ARG; ATA; ATG; BES; BHS; BLM; BLZ; BMU; BOL; BRA; BRB; BVT; CHL; COL; CRI; CUB; CUW; CYM; DMA; DOM; ECU; FLK; GLP; GRD; GTM; GUF; GUY; HND; HTI; JAM; KNA; LCA; MAF; MEX; MSR; MTQ; NIC; PAN; PER; PRI; PRY; SGS; SLV; SUR; SXM; TCA; TTO; URY; VCT; VEN; VGB; VIR
Middle East Asia	MEA	ARE; BHR; DZA; EGY; ESH; IRN; IRQ; ISR; JOR; KWT; LBN; LBY; MAR; OMN; PSE; QAT; SAU; SDN; SYR; TUN; YEM
Non-European Countries	NEU	ALB; AND; BIH; CHE; GRL; ISL; LIE; MCO; MKD; MNE; NOR; SJM; SMR; SRB; TUR; VAT
Other Asian Countries	OAS	AFG; ASM; ATF; BGD; BRN; BTN; CCK; COK; CXR; FJI; FSM; GUM; IDN; IOT; KHM; KIR; KOR; LAO; LKA; MDV; MHL; MMR; MNG; MNP; MYS; NCL; NFK; NIU; NPL; NRU; PAK; PCN; PHL; PLW; PNG; PRK; PYF; SGP; SLB; THA; TKL; TLS; TON; TUV; UMI; VNM; VUT; WLF; WSM
Reforming Economies	REF	ARM; AZE; BLR; GEO; KAZ; KGZ; MDA; RUS; TJK; TKM; UKR; UZB
Sub-Saharan Africa	SSA	AGO; BDI; BEN; BFA; BWA; CAF; CIV; CMR; COD; COG; COM; CPV; DJI; ERI; ETH; GAB; GHA; GIN; GMB; GNB; GNQ; KEN; LBR; LSO; MDG; MLI; MOZ; MRT; MUS; MWI; MYT; NAM; NER; NGA; REU; RWA; SEN; SHN; SLE; SOM; SSD; STP; SWZ; SYC; TCD; TGO; TZA; UGA; ZAF; ZMB; ZWE
United States of America	USA	USA

Supplementary Table 2: Engineered wood demand in 2050 and 2100 for all engineered wood demand scenarios and all MAgPIE regions. % Demand is the percentage share of regional demand in global demand (summing up to 100%).

	2050					2100				
	%	$Mm^3\ yr^{-1}$				%	$Mm^3\ yr^{-1}$			
	Demand	BAU	10pc	50pc	90pc	Demand	BAU	10pc	50pc	90pc
CAZ	1	0	1	6	11	1	0	3	14	26
CHA	20	0	25	123	222	10	0	25	123	222
EUR	3	0	4	21	38	3	0	7	37	67
IND	17	0	21	105	188	20	0	49	244	440
JPN	0	0	0	2	4	0	0	0	2	4
LAM	8	0	10	51	91	6	0	15	74	133
MEA	8	0	10	51	92	9	0	22	111	199
NEU	1	0	1	7	12	1	0	3	13	23
OAS	14	0	17	87	156	15	0	37	187	337
REF	1	0	2	8	14	1	0	3	14	25
SSA	23	0	29	144	259	30	0	76	381	685
USA	3	0	4	18	33	4	0	10	48	87
Total	100	0	125	623	1121	100	0	250	1250	2249

References

[1] Jan Philipp Dietrich, Christoph Schmitz, Christoph Müller, Marianela Fader, Hermann Lotze-Campen, and Alexander Popp. "Measuring agricultural land-use intensity–A global analysis using a model-assisted approach". In: *Ecological Modelling* 232 (2012), pp. 109–118.

[2] Abhijeet Mishra, Florian Humpenöder, Jan Philipp Dietrich, Benjamin Leon Bodirsky, Brent Sohngen, Christopher PO Reyer, Hermann Lotze-Campen, and Alexander Popp. "Estimating global land system impacts of timber plantations using MAgPIE 4.3.5". In: *Geoscientific Model Development Discussions* (2021), pp. 6467–6494. DOI: https://doi.org/10.5194/gmd-14-6467-2021.
