# Supplementary material for: Land use change and carbon emissions of a transformation to timber cities
Source: Nat Commun. 2022 Aug 30;13:4889. doi: 10.1038/s41467-022-32244-w (PMC9427734; doi:10.1038/s41467-022-32244-w)
Supplement: Supplementary file 2 — Reporting Summary [file 41467_2022_32244_MOESM2_ESM.pdf]

nature portfolio

Corresponding author(s): Abhijeet Mishra

Last updated by author(s): Jun 14, 2022

Reporting Summary

Nature Portfolio wishes to improve the reproducibility of the work that we publish. This form provides structure for consistency and transparency in reporting. For further information on Nature Portfolio policies, see our Editorial Policies and the Editorial Policy Checklist.

Statistics

For all statistical analyses, confirm that the following items are present in the figure legend, table legend, main text, or Methods section.

n/a	Confirmed	
☒	☐	The exact sample size (*n*) for each experimental group/condition, given as a discrete number and unit of measurement
☒	☐	A statement on whether measurements were taken from distinct samples or whether the same sample was measured repeatedly
☒	☐	The statistical test(s) used AND whether they are one- or two-sided<br>*Only common tests should be described solely by name; describe more complex techniques in the Methods section.*
☒	☐	A description of all covariates tested
☒	☐	A description of any assumptions or corrections, such as tests of normality and adjustment for multiple comparisons
☐	☒	A full description of the statistical parameters including central tendency (e.g. means) or other basic estimates (e.g. regression coefficient) AND variation (e.g. standard deviation) or associated estimates of uncertainty (e.g. confidence intervals)
☒	☐	For null hypothesis testing, the test statistic (e.g. F, t, r) with confidence intervals, effect sizes, degrees of freedom and P value noted<br>*Give P values as exact values whenever suitable.*
☒	☐	For Bayesian analysis, information on the choice of priors and Markov chain Monte Carlo settings
☒	☐	For hierarchical and complex designs, identification of the appropriate level for tests and full reporting of outcomes
☒	☐	Estimates of effect sizes (e.g. Cohen's d, Pearson's r), indicating how they were calculated

Our web collection on statistics for biologists contains articles on many of the points above.

Software and code

Policy information about availability of computer code

Data collection	The scenario data analysed in this paper are computed with the open-source framework MAgPIE (v4.3.5)<br>MAgPIE model version 4.3.5 is available at https://doi.org/10.5281/zenodo.6653242<br>Results can be replicated using the scripts and model code archived at https://doi.org/10.5281/zenodo.6643301
Data analysis	Array of R libraries tailed to analyze MAgPIE ouputs at Potsdam Integrated Assessment Modelling (PIAM): https://github.com/pik-piam/<br>Main libraries used:<br>May All Data be Reproducible and Transparent (MADRaT) (v 1.93.6): https://doi.org/10.5281/zenodo.1115490<br>MadRat commons Input Data Library (v0.22.0): https://doi.org/10.5281/zenodo.3822009<br>Visualization using R package ggplot2 (v3.3.5): https://github.com/tidyverse/ggplot2

For manuscripts utilizing custom algorithms or software that are central to the research but not yet described in published literature, software must be made available to editors and reviewers. We strongly encourage code deposition in a community repository (e.g. GitHub). See the Nature Portfolio guidelines for submitting code & software for further information.

Data

Policy information about availability of data

All manuscripts must include a data availability statement. This statement should provide the following information, where applicable:

- Accession codes, unique identifiers, or web links for publicly available datasets
- A description of any restrictions on data availability
- For clinical datasets or third party data, please ensure that the statement adheres to our policy

Model code used for this research is available on https://doi.org/10.5281/zenodo.6653242

Data used to initialize the model comes from open-source data sources stated within the meta-data information of model input files
Model Initialization data and input files are made available on https://doi.org/10.5281/zenodo.6551229

Field-specific reporting

Please select the one below that is the best fit for your research. If you are not sure, read the appropriate sections before making your selection.

☐ Life sciences ☒ Behavioural & social sciences ☐ Ecological, evolutionary & environmental sciences

For a reference copy of the document with all sections, see nature.com/documents/nr-reporting-summary-flat.pdf

Behavioural & social sciences study design

All studies must disclose on these points even when the disclosure is negative.

Study description	Model-based analysis of substituting conventional building materials (concrete and steel) with engineered wood to house urban dwellers of the future and its impact on land-use emissions.
Research sample	No sample needed as we used a model to study the impact of engineered wood demand in context of land-use on a global scale across different socio-economic futures.
Sampling strategy	No sampling startegy applicable as no samples were selected (see Research sample explanation above)
Data collection	Data is collected from publicly available data sources. Data sources are stated within the meta-data information of model input files.
Timing	Data obtained from public sources is processed to provide relevant inputs to the model. No data collelction timing applicable. Data used in this research was processed on 2021-09-01. Timeframe of input data used in the model spans from 1995-2100.
Data exclusions	MAgPIE simulations for this study are for time period 1995-2100. Results where comparison from 2020 is made, data for time period 1995-2020 are excluded for meaningful presentation of results. Model inputs are prepared based on simulation time steps needed in the model. Model inputs not belonging to the simulation time steps in the model are excluded from the simulation.
Non-participation	No participants involved in the study
Randomization	Outcomes of this study are based on model simulations which do not involve randomization

Reporting for specific materials, systems and methods

We require information from authors about some types of materials, experimental systems and methods used in many studies. Here, indicate whether each material, system or method listed is relevant to your study. If you are not sure if a list item applies to your research, read the appropriate section before selecting a response.

Materials & experimental systems

n/a	Involved in the study	
☒	☐	Antibodies
☒	☐	Eukaryotic cell lines
☒	☐	Palaeontology and archaeology
☒	☐	Animals and other organisms
☒	☐	Human research participants
☒	☐	Clinical data
☒	☐	Dual use research of concern

Methods

n/a	Involved in the study	
☒	☐	ChIP-seq
☒	☐	Flow cytometry
☒	☐	MRI-based neuroimaging
